# Investigating the antimethanogenic effects of selected nitro-compounds on methane production, rumen fermentation, and methanogenic archaea *in vitro*

**DOI:** 10.1128/aem.01033-25

**Published:** 2025-11-28

**Authors:** Alejandro Castaneda, Nagaraju Indugu, Krishna Challa, Kapil Narayan, Alexa Johnson, Darko Stefanovski, Terry Webb, Xin Zhao, Dipti Pitta

**Affiliations:** 1 Department of Clinical Studies, School of Veterinary Medicine, University of Pennsylvania 6572, Kennett Square, Pennsylvania, USA; 2 Department of Animal Science, Faculty of Agricultural and Environmental Sciences, McGill University 151165, Sainte-Anne-de-Bellevue, Quebec, Canada; University of Nebraska-Lincoln, Lincoln, Nebraska, USA

**Keywords:** methane, methanogenic archaea, nitro-compounds, rumen microbiome

## Abstract

Enteric methane emissions (EME) cause adverse environmental effects and energy losses to the host. Given the potent antimethanogenic effects of the nitro compounds (NC) ethyl-nitroacetate (ENA), ethyl-2-nitropropionate (ENP), and nitrate (NO₃⁻), we hypothesized that they could effectively mitigate EME. Phase 1 determined dose responses of each NC, identified optimal doses, and evaluated their effects on gaseous composition, rumen fermentation, and bacterial-archaea populations. ENA and ENP tested at 8 and 16 mM inhibited CH_4_ production by 100%, whereas NO₃⁻ tested at 12 and 24 mM reduced CH_4_ production by 72%. However, the amount of spared H_2_ differed among NC, revealing differential effects on fermentation pathways. ENA increased propionate and butyrate concentrations at the expense of acetate, whereas ENP slightly reduced acetate and modestly increased propionate at 24 h post-incubation. NO₃⁻ acted as an alternative H_2_ sink without changing fermentation end products. The overall methanogenic community was not altered, but each NC differentially reduced the abundance of *Methanobrevibacter ruminantium* M1, *Methanosphaera stadtmanae*, and the methanogenic archaeon ISO4-H5. ENA greatly altered the bacterial profiles, followed by ENP and NO₃⁻. Phase 2 investigated the impact of NC (0–1.25 mM) on *M. stadtmanae* cultures at 24 and 48 h post-incubation. The findings aligned with phase 1, confirming reduced CH_4_ production and H_2_ flux dynamics. ENA and ENP inhibited *M. stadtmanae* growth and CH_4_ production at all tested doses, whereas NO₃⁻ was effective at concentrations above 0.75 mM. These results highlight distinct mechanisms for CH_4_ mitigation, warranting further studies on additional methanogenic species to refine mitigation strategies.

**IMPORTANCE:**

EME signify detrimental environmental impacts and constitutes an energy loss for the host. ENA, ENP, and NO₃⁻ showed distinct antimethanogenic effects, resulting in varied impacts on gas composition, rumen fermentation, and bacterial-archaea populations. ENA exerted the strongest and most direct inhibitory effect on methanogenesis, leading to notable changes in H_2_ and VFA accumulations and archaeal populations. Although ENP completely inhibited CH_4_ production, it resulted in low H_2_ accumulations, suggesting an indirect effect and a dose-dependent modulation of fermentation pathways. NO₃⁻ produced a moderate reduction in CH_4_ output by diverting H_2_ toward NH_3_ production while maintaining fermentation stability. *M. stadtmanae* cultures verified that ENA, ENP, and NO₃⁻ have distinct mechanisms of action, thereby affecting methanogenesis differently. These findings highlight the potential of nitro-compounds for CH_4_ reduction, underscoring the need for *in vivo* validation alongside detailed multi-omics analyses to fully understand their effects on rumen microbiota and metabolic networks.

## INTRODUCTION

Methane (CH₄) is a potent greenhouse gas (GHG) with approximately 30 times the global warming potential of carbon dioxide (CO₂) over a 100-year period (EPA, 2024). According to the Intergovernmental Panel on Climate Change (IPCC) (1), agriculture, forestry, and other land use collectively contribute about 22% of total GHG emissions, with enteric CH_4_ emissions (EME) from beef and dairy cattle representing a major source. Although enteric fermentation is a natural and essential process for feed digestion and energy extraction, it results in a daily energy loss of 2%–12% of the gross energy intake of adult cattle (2, 3). Therefore, EME not only pose environmental risks but also represent energy inefficiencies to animal productivity.

Efforts to mitigate EME have focused on management practices, genetic selection, and feed-based interventions, including the use of antimethanogenic compounds (4). Among these, synthetic inhibitors and electron acceptors such as 3-nitrooxypropanol (3-NOP) and nitrate (NO₃⁻) have shown promising mitigation potential by reducing EME up to 35% (5–8). Although the mechanisms and efficacy of NO₃⁻ as a methane inhibitor are well established, its inclusion here serves as a comparative control to evaluate the less-studied nitro-compounds such as ethyl-nitroacetate (ENA) and ethyl-2-nitropropionate (ENP), whose broader impacts on rumen fermentation and microbial dynamics remain insufficiently characterized. These two nitro-compounds (NC) have demonstrated exceptional in vitro efficacy by reducing CH₄ production by up to 99% (9–11). Although 3-NOP is the only NC that is commercially used to reduce CH₄ emissions in livestock species, other NCs, while capable of reducing CH₄ emissions by 100%, much less is known about their mode of action and their effects on fermentation and ruminal microbes.

Previous studies evaluating ENA, ENP, and NO₃⁻ have tested only a limited range of concentrations and typically assessed outcomes at a single time point (24 h post-incubation), which limits our understanding of their dose-by-hour effects and temporal dynamics. For instance, ENA reduced CH₄ by >93% at 12 mM without altering volatile fatty acid (VFA) accumulations (9), whereas at 3 and 9 mM, CH₄ was reduced by 97% and 98%, respectively, with H₂ production increasing 12-fold and 17-fold, respectively (11). At 12 µmol/mL, both ENA and ENP reduced CH₄ by 100%, decreased total gas production (TGP), but increased H₂ production, with ENA producing higher amounts of H₂ than ENP. Both ENA and ENP also reduced acetate and propionate, but ENA produced higher levels of butyrate compared with ENP (12). These studies highlight these NCs’ potential to reduce CH₄ production; however, optimal doses, time-dependent effects, and impacts on the rumen microbiome remain to be established.

Although methanogenic archaea represent only 0.3%–3.3% of the rumen microbiome (13, 14), they play a crucial role in removing H₂ and maintaining H₂ partial pressure thresholds between 0.1 and 5.7 Pa in the rumen (15, 16). These H₂ partial pressure thresholds vary by archaeal species. *Methanobrevibacter*, a hydrogenotroph, operates efficiently at a threshold of 5.7 Pa (17), requiring four H₂ molecules for CH₄ synthesis. *Methanosphaera*, a methylotroph, requires only one H₂ molecule and can function at a threshold of 1.0 Pa (15, 18). Although hydrogenotrophic methanogenesis is thermodynamically more favorable, low H₂ levels can reduce the Gibbs free energy (ΔG°’) of hydrogenotrophs more than the methylotrophs and thus potentially allow *Methanosphaera* to outcompete *Methanobrevibacter* under these conditions. Although it is speculated that *Methanosphaera* may have a substantial contribution to EME than previously thought and that it may remove more H₂ than *Methanobrevibacter* under low H₂ conditions, testing the effects of NCs on total and individual methanogenic archaea may shed light on the contribution of individual methanogenic archaea to the total EME.

Therefore, we hypothesized that the selected NCs (ENA, ENP, and NO₃⁻) would reduce CH₄ production without compromising rumen fermentation via dose-by-hour interactions. We further hypothesized that each NC would differentially impact individual methanogenic archaea and consequently methanogenesis in the rumen. This study aimed to (i) investigate dose-by-hour interactions of NCs on the gaseous composition, rumen fermentation, and bacterial-archaea populations using mixed rumen cultures; (ii) identify optimal NC doses for maximum CH₄ mitigation without negatively impacting ruminal fermentation; and (iii) assess antimethanogenic effects of NCs on *Methanosphaera stadtmanae* using pure cultures. These insights are essential for developing feed additives that effectively reduce EME while maintaining rumen functionality and animal productivity.

## MATERIALS AND METHODS

**Fig 1:**
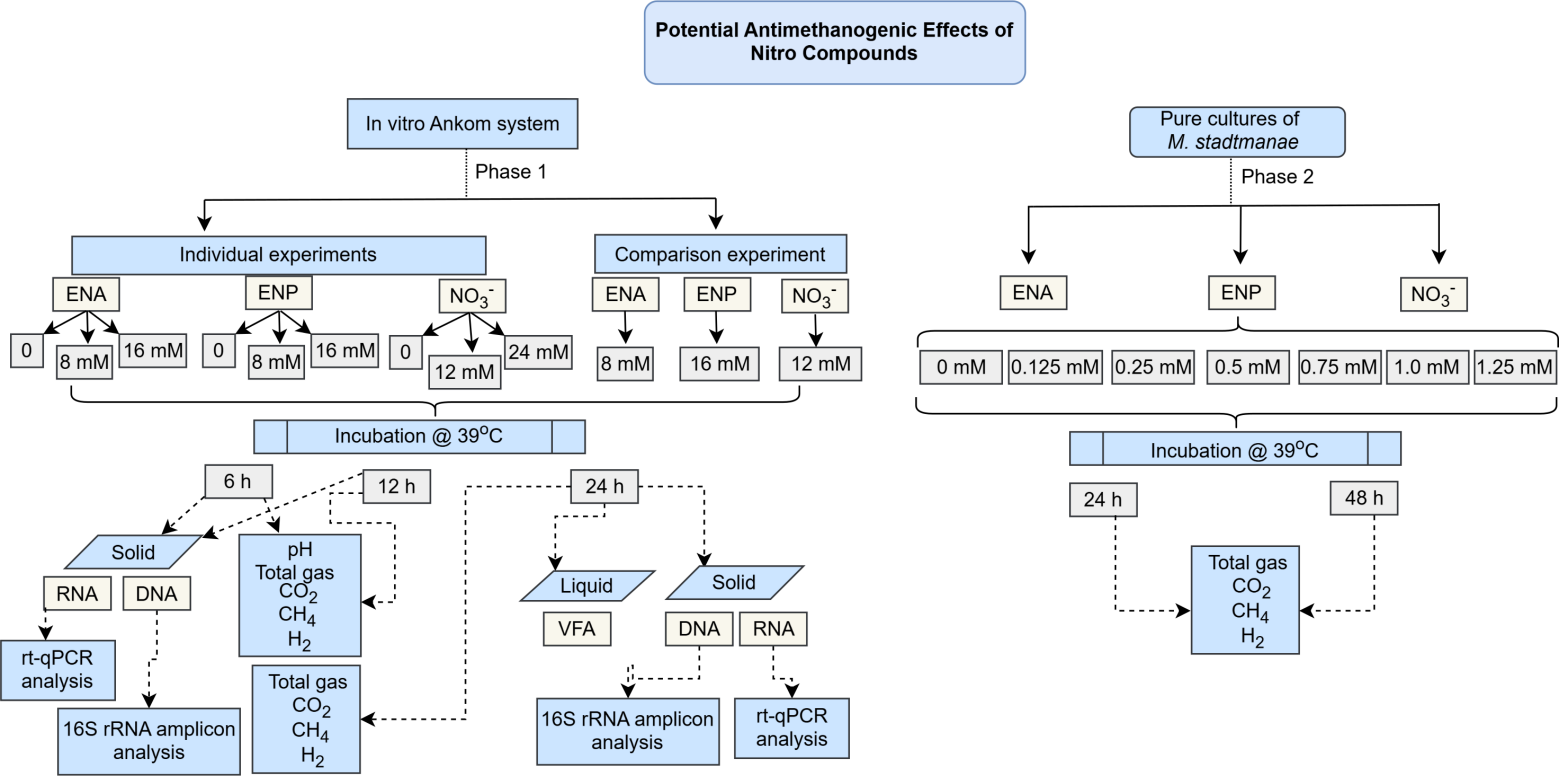
Experimental workflow for evaluating the antimethanogenic effects of NC.

This study involved two phases (Fig. 1). Phase 1 consisted of a series of *in vitro* experiments using the ANKOM RF Gas Production System (Ankom Technology, Macedon, NY). The aim was to evaluate the antimethanogenic effects of varying doses of NCs over different time points (dose-by-hour interactions) on TGP, gaseous composition, rumen fermentation parameters, and bacterial-archaea populations. For these purposes, ENA (97% purity; Alfa Aesar, Ward Hill, MA), ENP (96% purity; Thermo Scientific, Lancashire, UK), and calcium nitrate tetrahydrate (>99% purity; Thermo Scientific, Ward Hill, MA) were acquired. Consequently, an optimal dose for each NC was identified and compared in a combined ANKOM experiment. This phase used rumen fluid obtained from a single lactating donor cow, representing a mixed microbial population. Phase 2 focused on examining the dose-response relationship of each NC on optical density measured at a wavelength of 600 nanometers (OD_600_), TGP, and the production of individual gases (CO₂, CH₄, and H₂) using a pure culture of *M. stadtmanae in vitro*.

### Rumen fluid collection

In phase 1, a rumen-fistulated Holstein dairy cow, utilized as a donor of rumen fluid, was managed following the guidelines set forth by the University of Pennsylvania Institutional Animal Care and Use Committee (IACUC: Protocol number 80344). Rumen inoculum was collected from a single fistulated lactating Holstein cow maintained on a consistent total mixed ration throughout the study period. This animal was selected to ensure uniformity in microbial composition and physiological state across all experimental runs. The study involved multiple *in vitro* trials conducted over a 4-month period. To reduce variability introduced by factors such as parity, dry matter intake, production level, and stage of lactation on the ruminal microbiota, we used a single donor animal. This approach allowed for controlled, reproducible comparisons of multiple treatments and doses while minimizing inter-donor variation that could confound interpretation of microbial and fermentation responses. The donor cow was milked twice daily at 0400 and 1500 h, fed a total mixed ration (TMR) once daily at 0900 h, and had *ad libitum* access to drinking water. The TMR included grass hay (1.91%), protein source (29.7%), ground corn (16.7%), triticale (11.8%), and corn silage (39.9%). The donor cow was housed in a naturally ventilated free-stall housing system at The Marshak Dairy, University of Pennsylvania. All rumen fluid collections were performed at 0700 h. Whole rumen contents were collected from the cow’s feed mat, filtered on-site to remove particles > 5 mm, and brought to the laboratory within 20 min from collection. At the laboratory, the rumen fluid was poured into a 2.5 L beaker, flushed with 100% CO_2_ for 20 min at 39°C before mixing it with the buffer and dispensing the rumen inoculum to the vials.

### Nitro-compound selection and experimental workflow

#### Experiment 1

This preliminary experiment, whose results are not presented, aimed to determine doses and sampling hours at which the NC were assessed in subsequent experiments. The NCs were tested at 0, 8, and 16 mM, collecting samples at 6, 12, and 24 h post-incubation. This arrangement allowed observing the impact of varying doses on gaseous composition, rumen fermentation, and bacterial-archaea populations over time, given that gas production peaks between 12 and 24 h post-incubation. A total of 36 vials, or 9 vials per dose-by-hour combination, were filled with rumen inoculum and randomly assigned to each combination.

#### Experiments 2, 3, and 4

The aim was to test each NC in individual experiments to select the best-performing dose level out of 3 for each NC. ENA, ENP, and NO₃⁻ were assessed in experiments 2, 3, and 4, respectively. Based on the results obtained from experiment 1, ENA and ENP were tested at 8 and 16 mM, whereas NO₃⁻ was tested at 12 and 24 mM. These doses were selected because, according to experiment 1, NO₃⁻’s antimethanogenic effect at 8 and 16 mM was less potent than ENA and ENP at 8 and 16 mM; thus, NO₃⁻ doses were increased in experiment 4. In addition to these treatments, control (0 mM) and blanks were also included. A total of 108 vials, 36 per experiment or 9 per dose-by-hour combination (6, 12, and 24 h post-incubation), were filled with rumen inoculum and randomly assigned to each dose-by-hour combination. Bottles were sampled and terminated at each of their assigned sampling time.

#### Experiments 5 and 6

The best-performing dose of each NC was selected from experiments 2–4 and tested in experiments 5 and 6. The aim was to simultaneously test the best-performing doses selected from experiments 2–4 to choose the best NC and dose in experiments 5 and 6. In experiment 5, ENA, ENP, and NO₃⁻ were tested at 8, 16, and 12 mM, respectively. Control (0 mM) and blanks were also included. The experimental design of experiment 5 was duplicated in experiment 6 to validate the results obtained in experiment 5. A total of 225 vials, or 45 vials per experiment (6, 12, and 24 h post-incubation), were filled with rumen inoculum and randomly assigned to each treatment. Bottles were sampled and terminated at each of their assigned sampling time.

### *In vitro* gas production and preparation of the rumen inoculum

The ANKOM RF Gas Production System facilitated a series of *in vitro* experiments. Excluding blanks, 1.5 g of dried and ground TMR was added to 250 mL glass vials, along with 75 mL of freshly prepared McDougall buffer stabilized at pH 8.0 (19). Before setting each experiment, the buffer was brought to 39°C to prevent temperature shock to the rumen microbiome and mixed before dispensing 75 mL of rumen fluid into each vial for a total of 150 mL. Each vial was flushed with 100% CO_2_ through the Luer port for 30 s to ensure anaerobic conditions and then capped with modules (Ankom Technology, Macedon, NY). The incubation started by pressing “Record,” with a 5min recording interval, 1.5 psi for gas release, and 150 ms for open valves. The vials were randomly distributed inside the incubator (New Brunswick Excella E25 Inc incubator) to provide equal airflow across treatments, continuously shaken at 100 rpm/min, and incubated at 39°C for 24 h.

### Gaseous composition analysis

At each sampling hour, two 1 mL gas aliquots were collected from the vial’s headspace through the sampling port, as described in the ANKOM RF Operator’s Manual. Standards for CO_2_, CH_4_, and H_2_ at 100%, 100%, and 80% concentrations, respectively, were used to estimate the gaseous composition in the samples. Samples and standards were analyzed using an SRI 8610C BTU Gas Analyzer GC System (Bad Honnef, Germany) equipped with a Thermal Conductivity Detector (TCD), Flame Ionization Detector (FID), methanizer, and HayesSep D packed column (1.83 m × 2 mm). The TCD and FID were heated to 150°C and 300°C, respectively. Argon served as the carrier gas, flowing at 25 mL/min. At the end of each incubation, the gas pressure was converted from psi to mL to calculate TGP as instructed in the ANKOM manual. The obtained results were then used to estimate the composition of individual gases (mL).

### Rumen fermentation analysis

At each sampling hour, an unfiltered liquid sample was taken from each vial for pH measurement (Accumet AB150 pH meter; Fisher Scientific, Waltham, MA). The filtered liquid fraction of samples collected exclusively at 24 h was used to quantify the concentrations of total and individual VFA via high-performance liquid chromatography (HPLC). Briefly, the samples were thawed and centrifuged at 4,700 × *g* for 5 min at 4°C, and the supernatant of the triplicate vials was equally combined to make a single representative sample. The sample representatives were prepared as per Muck and Dickerson (1988), with the only deviation being that 25% sulfuric acid was utilized instead of full-strength sulfuric acid. The samples were filtered through a 0.22 µm filter into duplicate tubes for the analysis of acetic, propionic, butyric, isobutyric, valeric, and isovaleric acids. The analysis was performed in an Agilent 1260 Infinity II LC system HPLC with an Agilent Hi-Plex H (300 × 7.7 mm) column and a Refractive Index Detector, employing 0.005 M sulfuric acid as the mobile phase flowing at 0.6 mL/min. VFA concentrations were determined using standard curves for each metabolite.

### Microbial community analyses

To observe the impact of the NC on the bacterial populations, microbial community analyses were performed. At each sampling hour, whole vial contents were filtered through four layers of cheesecloth, yielding about 1.0 and 0.5 g of the solid fraction for DNA and RNA extraction, respectively. RNA samples were preserved in TRIzol. The genomic DNA was extracted from the solid fraction of the samples collected at 24 h post-incubation and then purified following the “Repeated Bead Beating plus Column” (RBB + C) method (20) using the QIAmp Fast DNA Stool Mini Kit (Qiagen Sciences; Germantown, MD, USA). The V1-V2 regions of the bacterial 16S rRNA gene were PCR-amplified in triplicate using the Accuprime Taq DNA Polymerase System (Invitrogen, Carlsbad, CA, United States) (21). The bacterial-specific primers used were F27 (5′-AGAGTTTGATCCTGGCTCAG-3′) and R338 (5′-TGCTGCCTCCCGTAGGAGT-3′). This primer pair was barcoded with a unique 12-base error-correcting Golay code for multiplexing (22). The thermal cycling conditions through which the samples were amplified were followed based on the methodology described by Pitta et al. (21). The triplicate amplicon products of each sample were pooled and then quantified using the Quantit-iT double-stranded DNA broad range assay kit (Invitrogen, Carlsbad, CA) and the Spectramax M2e microplate reader (Molecular Devices, San Jose, CA, USA). Finally, the generated amplicons of each sample were pooled in an equimolar concentration and then purified using Agencourt AMPure XP Beads (Beckman-Coulter; Indianapolis, IN, USA). The sequencing was conducted at the Children’s Hospital of Pennsylvania Microbiome Core of the University of Pennsylvania, using the MiSeq Illumina Platform (Illumina Inc., San Diego, CA, United States).

### RT-qPCR analysis

Afterward, the inhibitory effects of the NC on the total and individual methanogenic archaea populations were quantified using quantitative real-time PCR (RT-qPCR). The total RNA was extracted from the solid fraction via the TRIzol method and reverse transcribed to complementary DNA (cDNA) using the SuperScript VILO cDNA Synthesis Kit (Invitrogen) following the manufacturer’s protocol. The cDNA served as a template to amplify the V4-V5 regions of the 16S rRNA gene to quantify the log copy numbers of the total methanogenic archaea population, the *mtaB* gene to quantify *M. stadtmanae* (to represent methanol-utilizing methanogens), the *mtbB* gene to quantify methanogenic archaeon ISO4-H5 (to represent methylotrophic methanogenesis), and the *mcrG* gene to quantify *M. ruminantium* M1 (to represent hydrogenotrophic methanogenesis) (Table S1). A gradient PCR was performed using the AccuPrime Taq DNA Polymerase System (Invitrogen) according to the manufacturer’s instructions. The gradient PCR was performed by an initial denaturation at 94°C for 5 min, 30 cycles of denaturation at 94°C for 30 s, an annealing temperature range of 55°C–57°C for 90 s, extension at 72°C for 30 s, and a final extension at 72°C for 10 min. The quality of the PCR products was evaluated utilizing a 1.5% agarose gel electrophoresis and assessed visually in a BioRad Gel Doc XR^+^ Imaging System (23).

### Pure cultures utilizing *M. stadtmanae*

In phase 2, the *M. stadtmanae* DSM 3091 strain was obtained from the Deutsche Sammlung für Mikroorganismen und Zellkulturen (Braunschweig, Germany). Because no previous studies have been published reporting doses of the NC at which methanogenic archaea are inhibited using pure cultures, specifically for ENA and ENP, the study of Duin et al. (24) was used as a benchmark. Thus, the NCs were tested in a preliminary experiment at relatively low (0.1 mM) and high (3 mM) doses, and samples were collected at 24 and 48 h post-incubation.

After determining the doses, each NC (inhibitor levels) was tested at 0, 0.125, 0.25, 0.50, 0.75, 1.0, and 1.25 mM (dose levels), collecting samples at 24 and 48 h post-incubation (hour levels) in a 2 × 3 × 7 factorial design. A total of 126 tubes, 42 per experiment or 6 per hour-by-inhibitor-by-dose combination, were used. Each hour-by-inhibitor-by-dose combination was tested in triplicate using Hungate tubes, which were considered the experimental units.

The recipe for media preparation to culture *M. stadtmanae* is presented in Supplementary Materials S1. Each tube was flushed with 100% CO_2_ for 2 min, after which the medium was added. Five milliliters of the freshly prepared medium were dispensed into 16 × 125 mm screw thread style Hungate tubes. The tubes were then re-flushed for 1 min with 100% CO_2_, capped with flange-style butyl rubber stoppers, and screwed with caps provided with a 9 mm opening at the top for inoculation or sampling purposes. For effective sterilization, the tubes were autoclaved at 121°C for 45 min at 15 psi. After sterilization, each tube was decapped and flushed with H_2_:CO_2_ at an 80:20 (vol/vol; 1 bar) gas ratio for 2 min. Because methanol is a vital substrate that *M. stadtmanae* requires to grow, 100 µL was added at this step. Each tube was re-flushed with H_2_:CO_2_ at an 80:20 (vol/vol; 1 bar) gas ratio for 1 min. Although flushing, the NCs were then added to the tubes according to the specified doses. The tubes were then recapped and inoculated with 0.5 mL (10% of the final medium) of the fresh *M. stadtmanae* inoculum. The tubes were incubated in the dark at 39°C and shaken at 100 rpm.

At each sampling hour, the OD_600_ of all Hungate tubes was measured using a Spectronic 20D+ (Spectronic Instruments, Madison, WI) spectrophotometer to quantify the NC’s effect on the growth of *M. stadtmanae*. Subsequently, each tube’s gas pressure was measured using a needle pressure gauge, recorded in psi, and converted to mL to estimate each tube’s TGP. One milliliter of gas was extracted from each tube’s headspace to quantify amounts of CO_2_, CH_4_, and H_2_ through gas chromatography. Gas sample measurements and methods for gaseous composition analysis were the same as those used for the *in vitro* ANKOM RF Gas Production System.

### Statistical and bioinformatics analyses

The data were analyzed using SAS statistical software (version 9.4 TS1M8 [9.4 M8], SAS Institute Inc.). No statistical or bioinformatics analyses were performed on the blanks, as they were included for quality control purposes. Using the PROC UNIVARIATE procedure, a histogram was created to determine the distribution of the data. The histogram revealed that the data were not normally distributed and that a Poisson distribution was appropriate. Consequently, the Poisson distribution was applied to the data utilizing the “dist” and “link” functions of the PROC GLIMMIX procedure. Hour, inhibitor, and dose were treated as fixed effects. Post-hoc pairwise comparisons of least squares means (LSMeans) were performed using the Scheffé adjustment to identify significant differences among fixed effects and their interactions. Significant differences (*P* < 0.05) among LSMeans are indicated by different superscript letters in the tables. The response variables analyzed in phase 1 were TGP, CO_2_, CH_4_, and H_2_ accumulation, the log copy numbers of total and individual methanogenic archaea, pH, and total and individual VFA concentrations and molar proportions. The response variables analyzed in phase 2 were OD_600_, TGP, CO_2_, CH_4_, and H_2_ accumulation. α was set to 0.05, which was the cutoff point to determine statistical differences, where values < 0.05 were considered significant.

The bacterial 16S rRNA sequencing data were processed utilizing the QIIME2 version 2020.6 pipeline (22). The procedure encompassed sorting reads by sample through demultiplexing, trimming both forward and reverse reads to a length of 230 nucleotides, and merging paired reads. Unique DNA sequences, referred to as amplicon sequence variants (ASVs), were identified with the aid of the DADA2 plugin (25). Sequences underwent alignment using MAFFT (26), and a phylogenetic tree was constructed employing FastTree 2 (27). Taxonomic assignments were conducted by matching ASVs to the Greengenes reference database (v37) (28), utilizing a naive Bayes classifier (29). Alpha diversity, represented by observed ASVs, reflects the total number of taxa present, indicating community richness. The Shannon and Simpson indices, on the other hand, account for both richness and relative abundance, capturing overall diversity. Pielou’s evenness measures the uniformity of taxa distribution, highlighting dominance or balance within the community, as well as Beta diversity, indicated by both weighted (accounts for the abundance of taxa) and unweighted (considers only presence or absence of taxa) UniFrac metrics, were computed through the “qiime diversity” function after normalizing the data to a sample depth of 9,607 reads per sample. All plots were generated using R (v4.2.1) with the ggplot2 package (v3.5.1) for barplots (taxonomic composition) and scatterplots (PCoA); pheatmap (v1.0.12) for microbial abundance heatmaps; and corrplot (v0.95) for visualizing correlation matrices. Microbial community data were analyzed descriptively without applying formal statistical tests. Patterns in bacterial composition were visualized using heatmaps and diversity metrics to highlight trends rather than statistical significance.

## RESULTS

### Phase 1: Assessing dose-response effects and optimizing doses for the NC

#### Effect of the NC on gaseous composition

Dose-by-hour interactions were significant on TGP for ENP (*P* = 0.033) and NO₃⁻ (*P* = 0.041) only (Table 1). At 8 mM of ENP, TGP was significantly reduced at 12 h compared with both control and 16 mM of ENP, but the differences disappeared by 24 h. Overall, TGP decreased by 8.80% at 24 h, whereas at 16 mM of ENP, TGP decreased by 3.20% at 24 h relative to the 0 mM dose. Unlike ENP, TGP at 12 mM of NO₃⁻ increased by 6.10% at 24 h, whereas at 24 mM, TGP increased by 4.50% relative to the 0 mM dose.

**TABLE 1:** Antimethanogenic effects of different doses of NC on gas composition (mL), gas proportions (%), and pH across sampling hours^*a*^

Variable	Inhibitor^*b*^	6 h			12 h			24 h			SEM	*P*-value		
		Cntl	LD	HD	Cntl	LD	HD	Cntl	LD	HD		Dose	Hour	D x H
pH	ENA	6.24	6.22	6.22	5.95	5.97	6.02	5.79	5.90	5.90	0.02	0.024	<0.001	0.031
TGP (mL)		149	140	135	208	214	210	308	306	298	7.71	0.503	<0.001	0.789
CO_2_ (mL)		87.5	79.5	75.6	115	121	120	163	172	171	5.23	0.830	<0.001	0.307
CH_4_ (mL)		20.9^a^	0.01^b^	0.01^b^	37.7^a^	0.03^b^	0.03^b^	79.5^a^	0.27^b^	0.20^b^	0.88	<0.001	<0.001	<0.001
H_2_ (mL)		0.42^a^	7.06^ab^	16.9^b^	0.63^a^	54.4^b^	53.4^b^	1.03^a^	87.2^b^	80.8^b^	1.58	<0.001	<0.001	<0.001
pH	ENP	6.51	6.57	6.56	6.36	6.44	6.38	6.23^a^	6.32^b^	6.28^ab^	0.01	<0.001	<0.001	0.280
TGP (mL)		94.2	85.1	83.9	167^a^	131^b^	167^a^	250	228	242	5.36	<0.001	<0.001	0.033
CO_2_ (mL)		55.4	49.0	48.9	92.0	75.0	92.1	132^a^	156^b^	166^b^	2.81	0.001	<0.001	<0.001
CH_4_ (mL)		13.2^a^	0.01^b^	0.01^b^	30.4^a^	0.02^b^	0.02^b^	64.6^a^	0.03^b^	0.04^b^	0.44	<0.001	<0.001	<0.001
H_2_ (mL)		0.26^a^	2.55^b^	1.41^c^	0.50^a^	3.80^b^	4.97^c^	0.84^a^	6.55^b^	6.92^b^	0.18	<0.001	<0.001	<0.001
pH	NO₃⁻	6.23	6.23	6.16	6.06	6.08	6.00	5.85	5.83	5.78	0.01	<0.001	<0.001	0.581
TGP (mL)		165	160	160	231	223	227	312	331	326	4.21	0.869	<0.001	0.041
CO_2_ (mL)		97.0	96.9	100	127	129	132	165^ab^	188^a^	158^b^	4.17	0.052	<0.001	0.005
CH_4_ (mL)		23.1^a^	13.6^b^	13.4^b^	42.0^a^	22.2^b^	21.6^b^	80.8^a^	40.6^b^	37.9^b^	0.82	<0.001	<0.001	<0.001
H_2_ (mL)		0.46	0.33	0.33	0.70^a^	0.46^b^	0.47^b^	1.05^a^	0.68^b^	0.67^b^	0.02	<0.001	<0.001	<0.001

^*a*^ Data represent mean ± SEM of individual experiments. pH and gas composition values were derived from the ANKOM RF Gas Production System.

^*b*^ Doses: Cntl: 0 mM; LD: 8 mM (ENA, ENP) and 12 mM (NO₃⁻); HD: 16 mM (ENA, ENP) and 24 mM (NO₃⁻). Abbreviations: mM, millimolar; mL, milliliters; h, hour; TGP, total gas production; CO2, carbon dioxide; CH4, methane; H2, hydrogen; NC, nitro-compounds; ENA, ethyl-nitroacetate; ENP, ethyl-nitropropionate; NO₃⁻, nitrate; SEM, standard error of the mean. Within each hour, means in the same row that do not share a common superscript letter are significantly different (*P* < 0.05). The absence of superscript letters within a row indicates no significant differences.

Subsequently, the gaseous composition of the fermentation vials was analyzed, quantifying the amounts of CO_2_, CH_4_, and H_2_. CO_2_ proportions in untreated fermentation vials ranged between 53% and 59%, whereas in nitro-treated fermentation vials, they ranged between 49% and 69%. Although most gas contained in the vials’ headspace was quantified, the total gas quantified was ~71%, which was lower than the corresponding TGP for each treatment. Similar to TGP, dose-by-hour interactions were detected on CO_2_ production for ENP (*P* < 0.001) and NO₃⁻ (*P* = 0.005) only (Table 1). At 24 h, CO_2_ production significantly increased by 18.2% and 25.8% at 8 and 16 mM of ENP, respectively, relative to the 0 mM dose, which was not observed at either 6 h or 12 h. Likewise, at 24 h post-incubation, CO₂ production increased by 14% with the 12 mM NO₃⁻ treatment but did not differ from the control at 24 mM, compared with the 0 mM dose. As expected, all NCs exerted a potent suppressive effect on CH_4_ production, as reflected by the significant dose-by-hour interactions detected for ENA (*P* < 0.001), ENP (*P* < 0.001), and NO₃⁻ (*P* < 0.001) (Table 1). Notably, both ENA and ENP reduced CH_4_ production by >99% compared with the 0 mM dose across all incubation times, confirming their potent antimethanogenic effect. Similarly, NO₃⁻ also exhibited a marked inhibitory effect, reducing CH₄ production by 50% and 53% at 12 mM and 24 mM, respectively, after 24 h of incubation. Across all treatments, CH₄ proportions ranged from 0.01% to 12% in treated vials and from 14% to 26% in the untreated control. Similar to CH_4_ production, all NCs exerted a profound effect on H_2_ accumulation, detecting significant dose-by-hour interactions for ENA (*P* < 0.001), ENP (*P* < 0.001), and NO₃⁻ (*P* < 0.001) (Table 1). In perspective, accumulation was 98.8% and 98.7% higher at 24 h when ENA was added to the fermentation vials at 8 and 16 mM, respectively, compared with the 0 mM dose. Likewise, ENP also increased H_2_ accumulation, although at a smaller level. When ENP was added to the fermentation vials at 8 and 16 mM, H_2_ accumulation was increased by 10% across both doses at 24 h, respectively, relative to the 0 mM dose. Contrary to ENA and ENP, H_2_ accumulation in the fermentation vials treated with 12 mM and 24 mM of NO₃⁻ was 40% lower in both doses at all hours compared with the 0 mM dose. H_2_ proportions of all treated and untreated fermentation vials ranged between 0.20% and 29% and 0.28% and 0.34%, respectively.

#### Effect of the NC on rumen fermentation

The addition of the NCs to fermentation vials affected the pH. The dose-by-hour interaction significantly affected (*P* = 0.031) the pH of ENA-fermentation vials, which gradually decreased as the fermentation progressed (Table 1). Unlike ENA, the pH of ENP- and NO₃⁻-fermentation vials was significantly affected by the dose (*P* < 0.001) and hour (*P* < 0.001) effects, causing the pH to steadily decline during the incubation. Regarding VFA, the total concentration of VFA was not affected (*P* > 0.05) despite the addition of distinct NC at varying doses (Table 2). Acetate concentrations were significantly different among doses in ENA- (*P* < 0.001) and ENP-fermentation vials (*P* < 0.001) only (Table 2). Compared with the 0 mM dose, acetate accumulation declined by 24% and 21% in the fermentation vials treated with 8 and 16 mM of ENA at 24 h post-incubation, respectively. At 8 mM, ENP reduced acetate accumulation by 6% at 24 h post-incubation relative to the 0 mM dose. Propionate concentrations differed significantly among doses in ENA- (*P* < 0.001), ENP- (*P* < 0.001), and NO₃⁻-fermentation vials (*P* = 0.005) (Table 2). At 24 h post-incubation, propionate accumulations were 11% and 8% higher than the 0 mM dose when treated with 8 mM of ENA and 16 mM of ENP, respectively. At 12 mM and 24 mM, NO₃⁻ exerted a modest effect, increasing propionate accumulations by only 1% relative to the 0 mM dose. Only ENA caused butyrate accumulations to differ significantly (*P* = 0.009) among doses (Table 2), increasing by up to 19% with the 8 mM dose at 24 h post-incubation relative to the 0 mM dose. A dose effect was detected (*P* = 0.010) on valerate concentrations of vials treated with ENP (Table 2). Valerate concentrations were 10% higher with the 16 mM dose relative to the 0 mM dose at 24 h post-incubation. Isovalerate accumulations differed significantly (*P* = 0.002) among doses in ENA-fermentation vials. Isovalerate concentrations at 24 h post-incubation were 29% higher in vials treated with 8 mM of ENA compared with the 0 mM dose. Finally, isobutyrate accumulations differed significantly in ENA- (*P* < 0.001) and ENP-fermentation vials (*P* < 0.001) only (Table 2). Surprisingly, isobutyrate concentrations were 38% and 47% lower in vials treated with 16 and 8 mM of ENA and ENP at 24 h post-incubation, respectively, compared with the 0 mM dose.

**TABLE 2:** Antimethanogenic effects of different doses of NC on rumen fermentation parameters at 24 h of incubation^*a*^

VFA	ENA^*b*^			SEM	*P*-value	ENP			SEM	*P*-value	NO₃⁻			SEM	*P*-value
	Cntl	LD	HD			Cntl	LD	HD			Cntl	LD	HD		
Acetate (mM)	84.5^a^	64.3^b^	66.7^c^	0.65	<0.0001	55.0^a^	51.8^b^	55.4^a^	0.65	0.001	84.5^a^	83.5^b^	85.6^c^	0.65	0.005
Propionate (mM)	29.4^a^	32.5^b^	31.3^b^	0.28	0.010	22.4	23.6	24.3	0.16	0.094	24.9	25.1	25.0	0.15	0.946
Butyrate (mM)	25.5	30.3	26.6	0.37	0.077	14.7^a^	14.2^a^	16.7^b^	0.4	0.010	23.4	19.8	21.3	0.4	0.054
Valerate (mM)	3.26^a^	2.80^b^	2.80^b^	0.96	0.002	2.02	1.93	2.21	0.25	0.169	2.52	2.60	2.53	0.61	0.667
Isovalerate (mM)	4.35^a^	5.59^b^	4.70^c^	0.06	<0.0001	2.31^a^	2.33^a^	2.87^b^	0.08	0.000	3.57	3.47	3.61	0.06	0.321
Isobutyrate (mM)	1.88	1.45	1.17	0.02	0.219	1.17	0.62	1.18	0.02	0.153	1.45	1.62	1.51	0.03	0.884
Total VFA (mM)	149^a^	137^b^	133^b^	0.22	0.001	97.7^a^	94.5^b^	103^c^	0.17	0.003	140	136	140	0.23	0.063
Acetate (mol %)	56.7^a^	46.9^b^	50.2^c^	1.83	0.0002	56.3^a^	54.8^b^	53.8^b^	0.46	0.0040	60.4	61.4	61.1	0.28	0.1248
Propionate (mol %)	19.7^a^	23.7^b^	23.5^b^	0.83	0.0041	22.9^a^	25.0^b^	23.6^ab^	0.40	0.0438	17.8	18.5	17.9	0.17	0.2378
Butyrate (mol %)	17.1^a^	22.1^b^	20.0^ab^	0.96	0.0246	15.0	15.0	16.2	0.29	0.0431	16.7	14.6	15.2	0.44	0.0701
Valerate (mol %)	2.19	2.04	2.11	0.03	0.0752	2.07	2.04	2.15	0.04	0.6429	1.80	1.91	1.81	0.03	0.2311
Isovalerate (mol %)	2.92^a^	4.08^b^	3.53^c^	0.21	<0.0001	2.36^a^	2.47^a^	2.79^b^	0.08	0.0013	2.55	2.55	2.58	0.01	0.5231
Isobutyrate (mol %)	1.26	1.06	0.88	0.10	0.3717	1.20	0.66	1.15	0.13	0.1793	1.04	1.19	1.08	0.08	0.8137

^*a*^ Data represent mean ± SEM of individual experiments. Means within rows with different superscript letters differ significantly (*P *< 0.05). VFA concentrations were measured by an HPLC system.

^*b*^ Doses: Cntl: 0 mM; LD: 8 mM (ENA, ENP) and 12 mM (NO₃⁻); HD: 16 mM (ENA, ENP) and 24 mM (NO₃⁻). Abbreviations: mM, millimolar; mol %, molar proportion; VFA, volatile fatty acid; NC, nitro compounds; ENA, ethyl-nitroacetate; ENP, ethyl-2-nitropropionate; NO₃⁻, nitrate; SEM, standard error of the mean. Within each dose, means in the same row that do not share a common superscript letter are significantly different (*P* < 0.05). The absence of superscript letters within a row indicates no significant differences.

Regarding the molar proportions of VFAs, acetate proportions differed significantly among doses in ENA- (*P* = 0.0002) and ENP-treated vials (*P* = 0.004) but not in NO₃⁻-treated vials (*P* = 0.125) (Table 2). Compared with the 0 mM control, the molar proportion of acetate decreased by 17% and 11% in vials treated with 8 and 16 mM of ENA, respectively, whereas ENP caused smaller decreases of 3% and 4% at 8 and 16 mM, respectively. Propionate proportions were significantly affected by dose in ENA- (*P* = 0.004) and ENP-fermentation vials (*P* = 0.044) but not in NO₃⁻-treated vials (*P* = 0.238). At 24 h post-incubation, ENA increased propionate proportions by 20% and 19% at 8 and 16 mM, respectively, relative to the 0 mM control, whereas ENP increased propionate by 9% at 8 mM but only 3% at 16 mM.

Butyrate molar proportions differed significantly among doses in ENA- (*P* = 0.025) and ENP-treated vials (*P* = 0.043), increasing by 29% and 17%, respectively, at the high doses compared with the control. In NO₃⁻-treated vials, the butyrate proportion tended to decrease (*P* = 0.070). Valerate proportions were not significantly affected by any treatment (*P* > 0.05). Isovalerate proportions differed significantly among doses in ENA- (*P* < 0.001) and ENP-treated vials (*P* = 0.001) only, increasing by 40% with 8 mM ENA and by 18% with 16 mM ENP relative to the control. In contrast, isobutyrate proportions did not differ significantly across treatments (*P* > 0.05).

#### Effect of the NC on individual methanogenic archaea

The log copy numbers of the total populations of methanogenic archaea did not change (*P* > 0.05) in any of the fermentation vials, either treated or untreated (Table 3). However, the log copy numbers of the individual populations of methanogenic archaea varied due to the influence of the NC. The log copy numbers of *M. stadtmanae* in ENA (*P* = 0.006), ENP (*P* < 0.001), and NO₃⁻-treated cultures (*P* = 0.006) were significantly affected by the dose-by-hour interaction (Table 3). The 8 mM dose of ENA reduced the log copy numbers of *M. stadtmanae* by 6% relative to the 0 mM dose, particularly at the 12 h incubation. Similarly, the log copy numbers of *M. stadtmanae* also significantly decreased with 12 mM of NO₃⁻ at 12 h. Although non-significant, the log copy numbers of *M. stadtmanae* at 24 mM of NO₃⁻ were reduced by 4% compared with the 0 mM dose, respectively. The log copy numbers of *M. ruminantium* M1 differed significantly in ENA- (*P* < 0.001) at all hours of incubation, except at 12 h, where 16 mM of ENA significantly increased M. ruminantium. Although both doses of NO₃⁻ lowered the log copy numbers of M. ruminantium at 6 and 12 h (*P* < 0.001), the effect diminished by 24 h (Table 3). At 24 h post-incubation, ENA and NO₃⁻ reduced the log copy numbers of *M. ruminantium* M1 by 10% and 4% when administered at 8 and 12 mM, respectively. Similar to *M. stadtmanae*, the log copy numbers of the methanogenic archaeon ISO4-H5 were significantly reduced in the high dose of ENA at 6 and 12 h (*P* < 0.001), but the effect diminished with incubation time. At 24 h post-incubation, the log copy numbers were 19% and 4% higher when ENA was added at 8 and 16 mM, respectively, compared with the 0 mM dose (Table 3). Although ENP did not have any effects on methanogenic archaeon ISO4-H5, NO₃⁻ lowered the log copy number at 6 h with the high dose, but the effect faded away with incubation time. When NO_3_^-^ was added at 12 mM and 24 mM, the log copy numbers were 8% and 5% higher at 24 h post-incubation, respectively, relative to the 0 mM dose.

**TABLE 3:** Antimethanogenic effects of different doses of NC on methanogenic archaea populations quantified by RT-qPCR across sampling hours^,,^

Methanogen (genes)^*a*^	Inhibitor^*b*^	6 h			12 h			24 h			SEM	*P*-value		
		Cntl	LD	HD	Cntl	LD	HD	Cntl	LD	HD		Dose	Hour	D x H
Total methanogens (16S rRNA)	ENA	15.9	15.6	15.6	15.5	15.9	15.5	15.8	15.5	15.8	0.14	0.346	0.243	0.637
*M. stadtmanae* (*mtaB*)		4.92	4.62	4.66	5.31^a^	4.8^b^	5.29^a^	5.13	4.82	4.86	0.06	<0.001	<0.001	0.006
*M. ruminantium* M1 (*mcrG*)		6.57^a^	4.94^b^	4.88^b^	6.64^a^	5.26^b^	5.46^b^	6.27^a^	5.65^b^	6.36^a^	0.08	<0.001	<0.001	<0.001
Archaeon ISO4-H5 (*mtbB*)		3.71^a^	3.55^a^	2.31^b^	3.6^a^	3.7^a^	2.56^b^	3.37	4.02	3.51	0.12	<0.001	<0.001	<0.001
Total methanogens (16S rRNA)	ENP	15.8	15.5	15.5	15.6^ab^	15.8^a^	15.6^b^	15.7	15.4	15.9	0.09	0.544	0.344	0.536
*M. stadtmanae* (*mtaB*)		4.70	4.78	4.83	5.14^a^	5.11^a^	4.80^b^	4.70^ab^	4.93^a^	4.60^b^	0.04	<0.001	<0.001	<0.001
*M. ruminantium* M1 (*mcrG*)		6.72	6.55	6.37	6.83	6.65	6.67	6.91	6.91	6.65	0.07	<0.001	<0.001	0.206
Archaeon ISO4-H5 (*mtbB*)		4.33	4.33	3.50	4.10	4.31	4.74	4.36	4.35	4.58	0.23	0.935	0.119	0.035
Total methanogens (16S rRNA)	NO₃**⁻**	15.7	15.6	15.4	15.7	15.7	15.7	15.6	15.5	15.8	0.10	0.643	0.245	0.437
*M. stadtmanae* (*mtaB*)		4.37	4.25	4.30	4.78^a^	4.5^b^	4.6^ab^	4.47	4.43	4.28	0.03	<0.001	<0.001	0.006
*M. ruminantium* M1 (*mcrG*)		7.14^a^	6.22^b^	6.10^b^	7.23^a^	6.44^b^	6.55^b^	7.08	6.77	7.00	0.06	<0.001	<0.001	<0.001
Archaeon ISO4-H5 (*mtbB*)		4.02^a^	3.94^a^	2.90^b^	3.85	4.00	3.65	3.87	4.19	4.05	0.14	<0.001	0.006	0.003

^*a*^ Data is expressed as log10 copies per 100 ng cDNA (mean ± SEM) from individual experiments.

^*b*^ Doses: Cntl: 0 mM; LD: 8 mM (ENA, ENP) and 12 mM (NO₃⁻); HD: 16 mM (ENA, ENP) and 24 mM (NO₃⁻). Abbreviations: Cntl: Control; LD: Low Dose; HD: High Dose; mM, millimolar; h, hour; D, dose; H, hour; M. stadtmanae, Methanosphaera stadtmanae; M. ruminantium M1, Methanobrevibacter ruminantium; NC, nitro-compound; ENA, ethyl-nitroacetate; ENP, ethyl-2-nitropropionate, NO₃⁻, nitrate; SEM, standard error of the mean. Within each hour, means in the same row that do not share a common superscript letter are significantly different (*P* < 0.05). The absence of superscript letters within a row indicates no significant differences.

### Microbial diversity and community composition analysis

**Fig 2:**
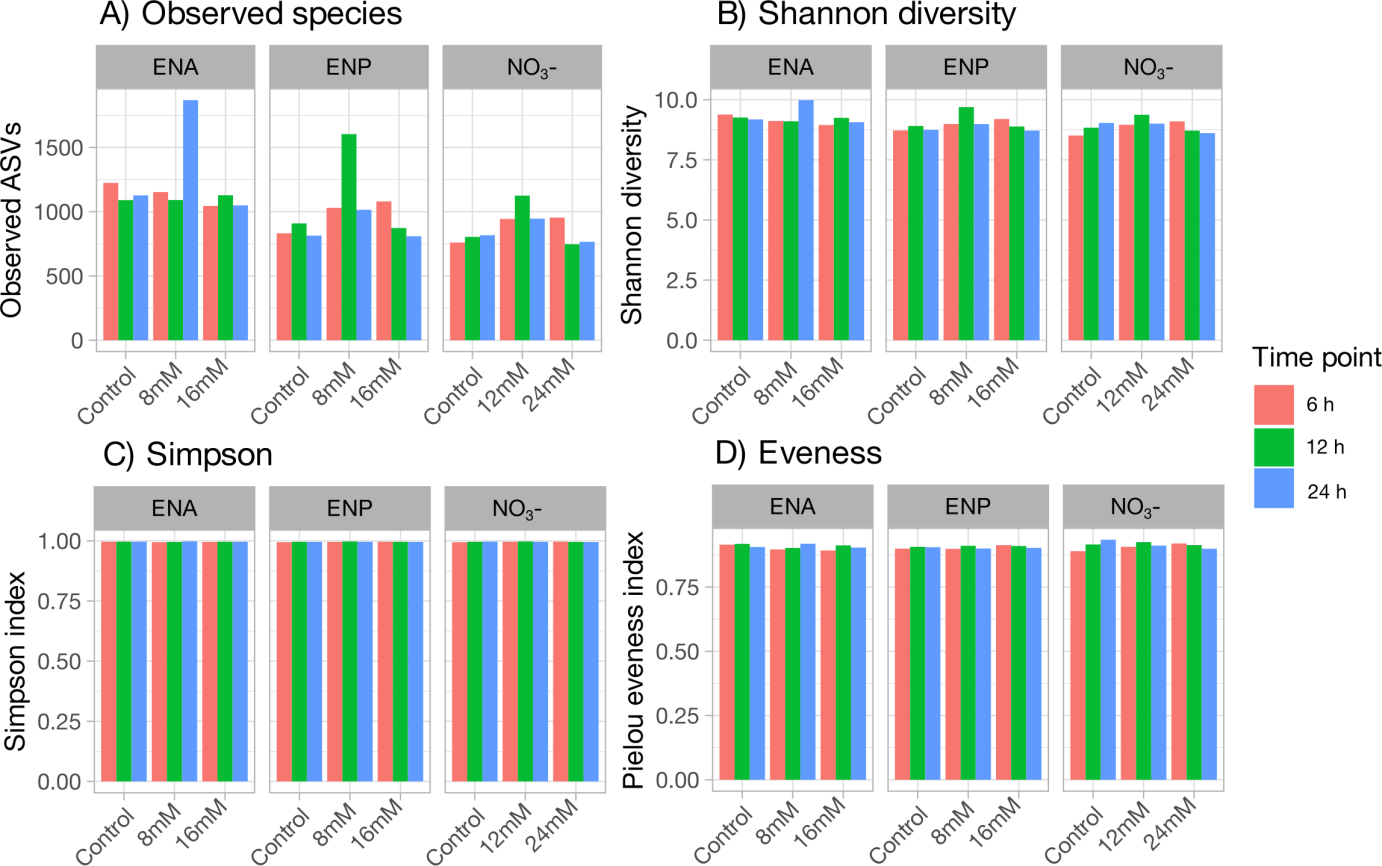
Effects of NC on rumen bacterial alpha diversity. (**A**) Observed ASV (amplicon sequence variants) richness, (**B**) Shannon diversity index, (**C**) Simpson diversity index, and (**D**) Pielou’s evenness across treatments. Treatments included ENA (8 and 16 mM), ENP (8 and 16 mM), and NO₃⁻ (12 and 24 mM) at sampling 6, 12, and 24 h.

For alpha diversity, observed amplicon sequence variants (ASV), Shannon diversity index, and Simpson and Pielou’s evenness were assessed to compare microbial profiles among NC, doses, and hours (Fig. 2). In ENA-fermentation vials, the observed ASV at 8 mM at 24 h was higher than at 6 and 12 h, nearly double the ASV in the 0 and 16 mM doses. In ENP-fermentation vials, the observed ASV at 8 mM at 12 h was the highest, exceeding the 0 and 16 mM doses. The observed ASV in the 16 mM dose at 6 h exceeded that at 12 and 24 h (Fig. 2A). In NO₃^-^-fermentation vials, ASV at 8 mM at 12 h was higher than that at 6 and 24 h, exceeding both 0 and 24 mM doses. ASV at 24 mM at 6 h surpassed that at 12 and 24 h, including the 0 mM dose (Fig. 2A). The Shannon diversity index in ENA-fermentation vials at 8 mM at 24 h was the highest, surpassing the 0 and 16 mM doses. In ENP-fermentation vials, species diversity peaked at 8 mM at 12 h, higher than the 0 and 16 mM doses. Diversity at 16 mM at 6 h was higher than that at 12 and 24 h. Species diversity in NO₃⁻-fermentation vials at 12 mM at 12 h exceeded that at 6 and 24 h, surpassing 0 and 24 mM doses. Diversity at 24 mM at 6 h was higher than at 12 and 24 h, surpassing the 0 mM dose (Fig. 2B). No visual differences were noted in Simpsons and Pielou’s evenness metrics between treatments.

**Fig 3:**
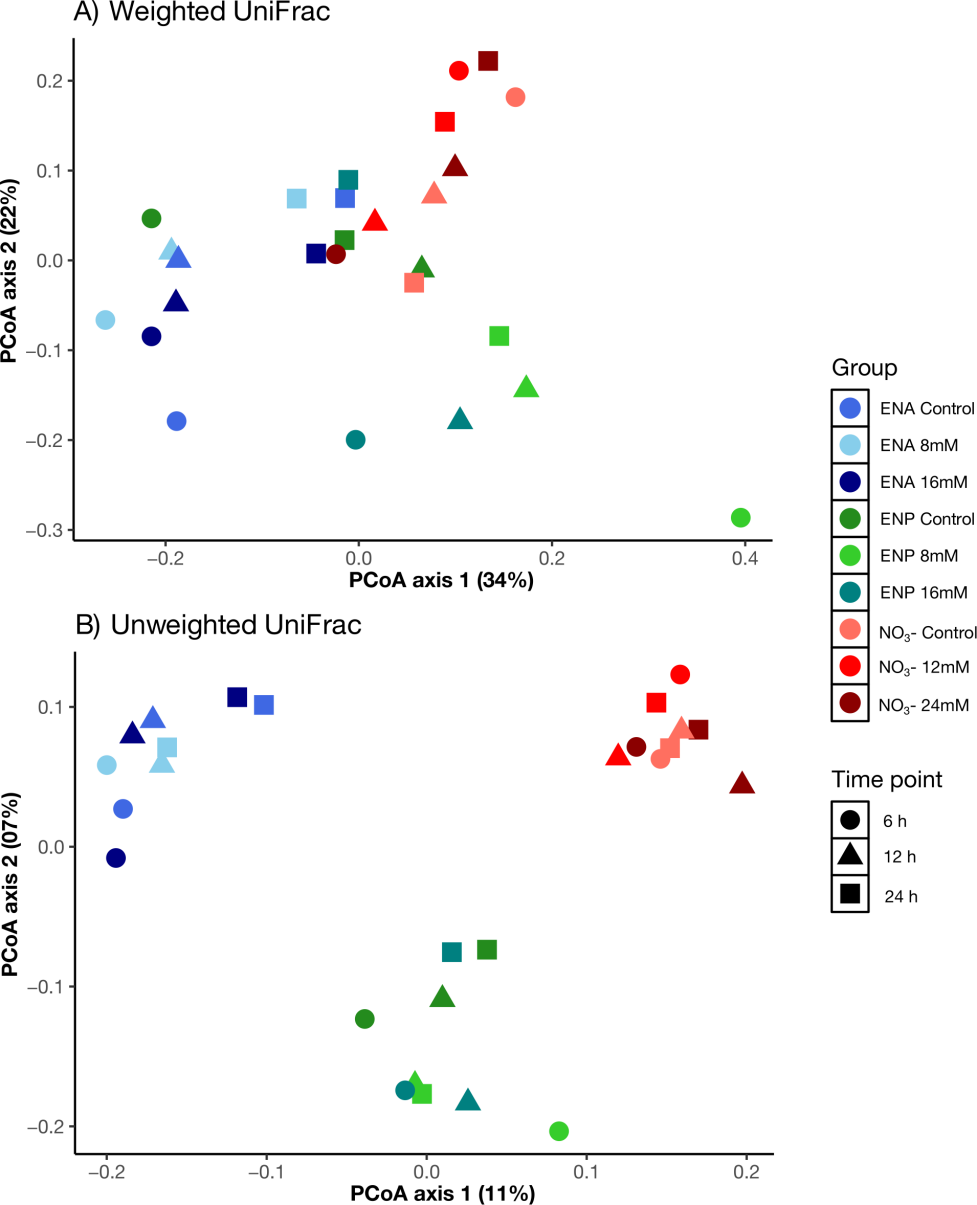
The effects of NC on bacterial community composition were analyzed using principal coordinate analysis (PCoA) based on (**A**) weighted and (**B**) unweighted UniFrac distances. Treatments included ENA (8 and 16 mM), ENP (8 and 16 mM), and NO₃⁻ (12 and 24 mM) at sampling hours 6, 12, and 24 h.

Figure 3 shows the principal coordinates analysis (PCoA) based on weighted and unweighted UniFrac distances to evaluate microbial community differences across treatment groups and time points. In weighted UniFrac (Fig. 3A), the first two axes explain 34% and 22% of the variation, respectively (totaling 56%). Despite the higher variance explained, there is limited clustering among the treatment groups, suggesting that differences in microbial structure based on relative abundance are subtle. In contrast, unweighted UniFrac (Fig. 3B) explains less overall variation (11% and 7% on axes 1 and 2, respectively); however, it shows clearer clustering by treatment levels and time points. These findings indicate that changes in microbial community membership (i.e., presence or absence of taxa) are more distinct than changes in relative abundance across these treatment conditions.

**Fig 4:**
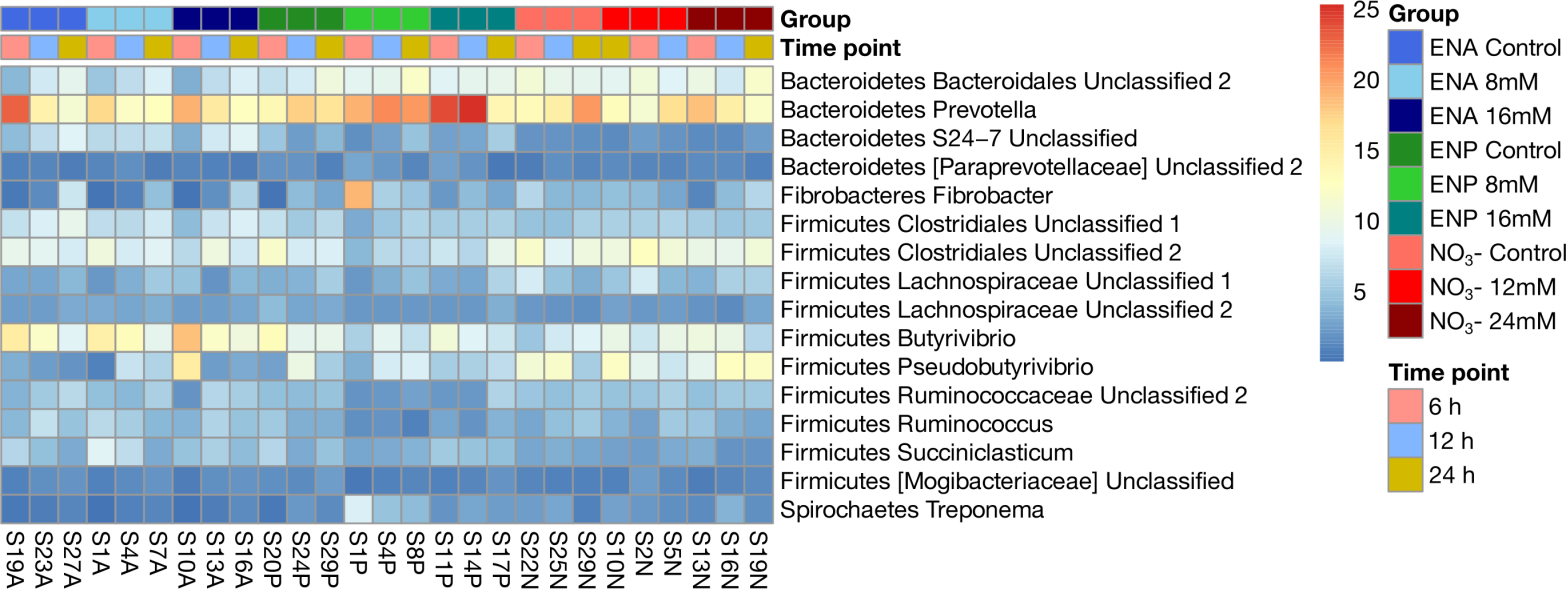
Heatmap of bacterial genus-level relative abundances (%) across NC and sampling hours. Rows represent bacterial genera, and columns represent samples grouped by treatment and sampling hour. Treatments included ENA (8 and 16 mM), ENP (8 and 16 mM), and NO₃⁻ (12 and 24 mM) at sampling hours 6, 12, and 24 h. Sample IDs are labeled as follows: “S#A” indicates ENA treatment, “S#P” indicates ENP treatment, and “S#N” indicates NO₃⁻ treatment, where “S#” refers to the sample number.

Variations in the bacterial profiles among NC, doses, and hours were observed (Fig. 4; Table S2). In ENA-fermentation vials, the relative abundance of 22 bacterial genera, including Bacteroidetes *Bacteroidales Unclassified 2*, Bacteroidetes *BF311*, and Fibrobacteres *Fibrobacter*, fluctuated based on doses and hours. Notably, the abundance of Bacteroidetes *Bacteroidales Unclassified 2* decreased at 8 and 16 mM compared with the 0 mM dose, with a more significant reduction at 16 mM. This suggests a dose-dependent response, as Bacteroidetes *Bacteroidales Unclassified 2* appears susceptible to higher doses of ENA. Contrarily, Firmicutes [*Mogibacteriaceae*] Unclassified showed higher abundance in ENA vials than in non-treated ones, peaking at 16 mM compared with 8 mM. In ENP-treated vials, 16 bacterial genera, including Bacteroidetes *BS11 Unclassified*, Firmicutes *Lachnospiraceae Unclassified 1*, and Firmicutes *Pseudobutyrivibrio*, displayed shifts across doses and hours. The relative abundance of Bacteroidetes *BF311* increased from 27% to 55% compared with the 0 mM dose, although this effect was more pronounced at 8 mM than at 16 mM. The abundance of Bacteroidetes *Bacteroidales Unclassified 1* was lower at 16 mM than at 8 mM. In NO₃^-^-fermentation cultures, 15 bacterial genera, such as Bacteroidetes *Prevotella*, Bacteroidetes *YRC22*, and Firmicutes *Christensenellaceae Unclassified*, showed variations. The abundance of Firmicutes *Anaerovibrio* was lower at 24 mM than at 12 mM at both 6 and 24 h post-incubation, along with Firmicutes *Bulleidia* and Firmicutes *L7A_E11*. Conversely, Bacteroidetes *CF231* increased at 12 mM and 24 mM doses, especially at 6 and 12 h post-incubation. Overall, ENA caused the most changes in the bacterial profiles, followed by ENP and NO₃⁻.

### Correlations between VFA and bacterial taxa

**Fig 5:**
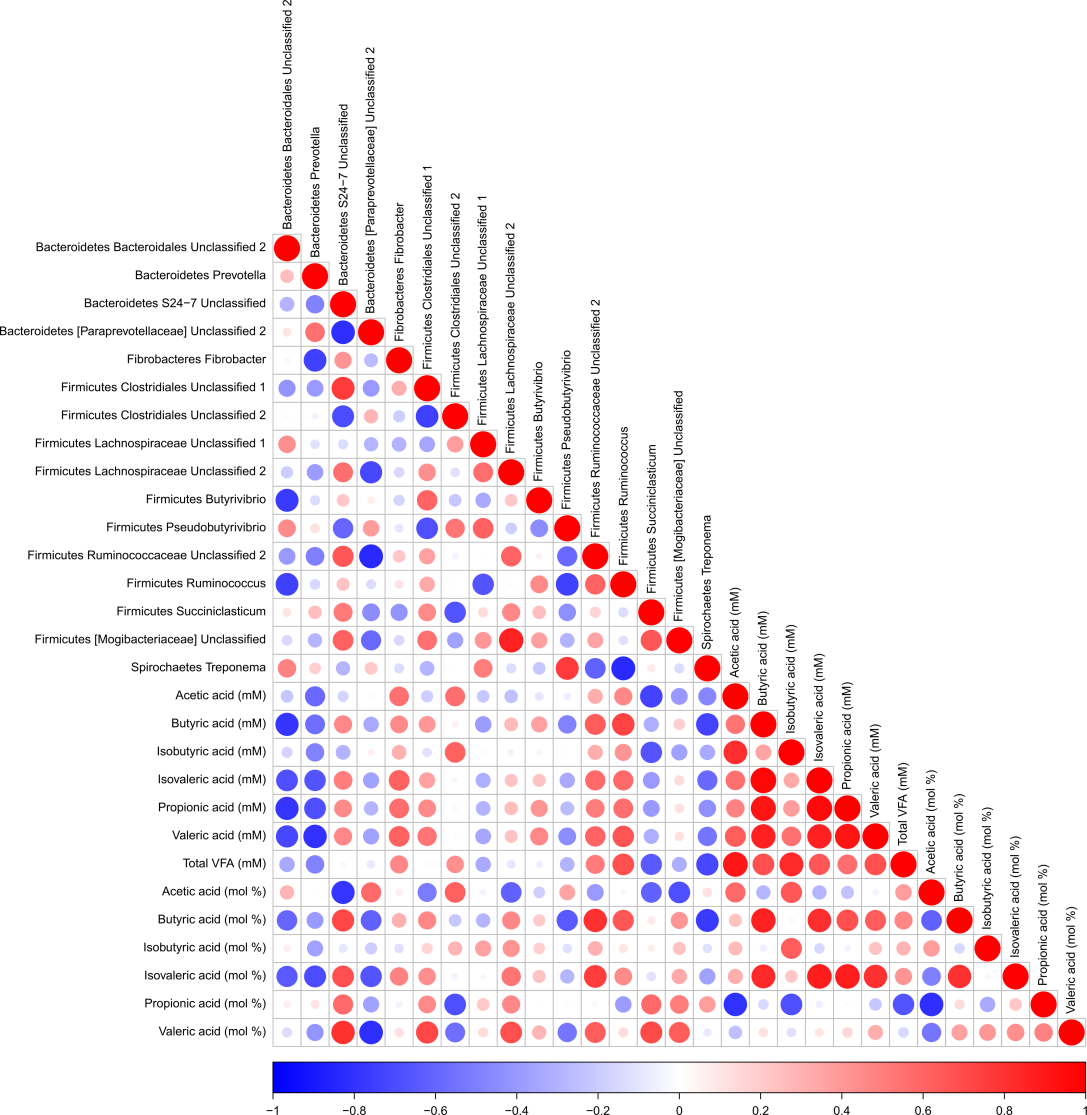
Spearman rank correlations between rumen bacterial genera and fermentation profiles (mM concentrations and molar %). Positive correlations (light to dark red) and negative correlations (light to dark blue) are indicated by color intensity. VFA = volatile fatty acids.

Spearman correlations between VFA and the most abundant bacterial genera are shown in Fig. 5 Tables S3 and S4. The results revealed that strong inverse correlations were detected between Bacteroidetes *Bacteroidales Unclassified 2* and butyrate (*P* = 0.013; R = −0.78), isovalerate (*P* = 0.042; R = −0.68), propionate (*P* = 0.013; R = −0.78), and valerate (*P* = 0.030; R = −0.72). Bacteroidetes *Prevotella* inversely associated with propionate (*P* = 0.042; R = −0.68), valerate (*P* = 0.010; R = −0.80), and isovalerate (*P* = 0.050; R = −0.67). A strong inverse association was also found between Spirochaetes *Treponema* and butyrate (*P* = 0.025; R = −0.73). Firmicutes *Succiniclasticum* exhibited a strong inverse relationship with acetate (*P* = 0.025; R = −0.73) and isobutyrate (*P* = 0.050; R = −0.67). Conversely, Firmicutes *Ruminococcus* showed a strong positive association with butyrate (*P* = 0.025; R = 0.73) and valerate (*P* = 0.050; R = 0.80).

### Comparison of NC on gaseous composition, rumen fermentation, and methanogenic archaeal populations

Based on the findings from the individual inhibitor trials (experiments 2–4), optimal doses for the combination experiment were selected to balance methane inhibition with fermentation stability. For ENA, the low dose (8 mM) was chosen over the high dose (16 mM) since, despite nearly complete suppression of CH₄ at 16 mM, the lower dose maintained higher H₂, TVFA, and butyrate concentrations, suggesting fewer adverse effects on fermentation. For ENP, the high dose (16 mM) was selected because it yielded greater TGP, spared H₂, and supported higher TVFA and butyrate levels compared with the low dose. For NO₃⁻, 12 mM was identified as the lowest effective dose that reduced CH₄ by 72% while sustaining fermentation and avoiding excessive NO₂ accumulation, thereby providing a suitable balance between efficacy and safety. The doses selected for each inhibitor for the combination experiments were 8 mM of ENA, 16 mM of ENP, and 12 mM of NO_3_^-^, doses that were compared with a control using rumen fluid from a single donor cow to determine whether the findings were repeatable in experiments 5 and 6, and to compare the antimethanogenic potential of the selected NCs. Notably, the observed results for the respective doses selected from the individual experiments were replicated in a combined experiment, obtaining similar results (Table 4), thus offering greater confidence in the repeatability of the results and the experimental setup performed. At 24 h, the TGP was significantly lower (*P* < 0.01) in ENP compared to the control only, but not with either ENA or NO₃⁻ treatments. On the gaseous composition, while there are no differences in CO_2_ concentrations, ENA and ENP reduced CH_4_ production by > 99% at all hours, although NO₃⁻ reduced CH_4_ production by only 12.5% at 24 h relative to the control (Table 4). Moreover, H_2_ accumulation in ENA and ENP was 99% and 88%, whereas in NO₃⁻-fermentation vials, it was 40% higher at 24 h, respectively, relative to the 0 mM dose (Table 4), similar to the findings of individual experiments. However, the H2 accumulation was significantly higher in ENA compared with all other treatments. Regarding rumen fermentation, the findings obtained in the individual experiments agree with the results collected in the combined experiment (Experiment 5), in which the pH in the fermentation vials declined while the incubation was in progress (Table 4). Notably, at 24 h, pH differed among treatments, being the highest in ENA-treated and the lowest in ENP-treated vials.

**TABLE 4:** Antimethanogenic effects of different doses of NC on: (i) gas parameters (composition [mL], proportions [%]), and (ii) methanogen archaea abundance (total methanogenic archaea copies and strain-specific quantification) across sampling hours^*a*^

Variable	6 h				12 h				24 h				SEM	*P*-value	
	Cntl	ENA	ENP	NO₃⁻	Cntl	ENA	ENP	NO₃⁻	Cntl	ENA	ENP	NO₃⁻		Inhibitor	Hour
pH	6.24	6.27	6.28	6.25	6.04	6.12	6.05	6.07	5.83^ab^	5.95^a^	5.78^b^	5.83^ab^	0.02	<0.001	<0.001
TGP (mL)	106	93.4	97.3	106	152	138	156	153	218^a^	201^ab^	158^b^	225^a^	7.35	<0.001	<0.001
CO_2_ (mL)	62.2	59.1	66.7	65.6	83.6	77.9	85.9	88.8	107	103	109	109	4.24	0.225	<0.001
CH_4_ (mL)	14.8^a^	0.01^b^	0.01^b^	15.2^a^	27.6^a^	0.02^b^	0.02^b^	28.3^a^	56.6^a^	0.18^b^	0.02^b^	49.6^c^	1.53	<0.001	<0.001
H_2_ (mL)	0.30	4.71	1.65	0.22	0.46^a^	24.6^b^	3.84^a^	0.32^a^	0.73^a^	52.7^b^	4.52^a^	0.46^a^	0.91	<0.001	<0.001
Total methanogens (16S rRNA)	15.6	15.7	15.3	15.8	15.7	15.6	15.8	15.1	15.5^a^	15.7^b^	15.9^b^	15.7^b^	0.13	0.007	0.207
*M. stadtmanae* (mtaB)	3.95	4.15	3.71	3.77	3.56^ab^	3.50^ab^	4.14^a^	3.19^b^	3.50^a^	4.45^b^	3.72^ab^	3.89^ab^	0.12	0.001	0.002
*M. ruminantium* M1 (mcrG)	6.58^a^	4.16^b^	6.19^a^	6.48^a^	6.11^a^	4.32^b^	6.51^a^	6.56^a^	6.56^a^	4.93^b^	6.43^a^	6.14^a^	0.07	<0.001	0.008
Archaeon ISO4-H5 (mtbB)	4.41	4.43	3.79	4.45	4.44	4.30	4.49	4.48	4.85	5.02	4.42	4.85	0.12	0.002	<0.001

^*a*^ Data represent mean ± SEM from the comparison experiment. The selected doses represent optimal concentrations from the initial screening: 8 mM (ENA), 16 mM (ENP), and 12 mM (NO₃⁻). Gas composition values (mL) were derived from the ANKOM RF Gas Production System. Total and strain-specific methanogen abundance was quantified by RT-qPCR (log10 copies/100 ng cDNA). Abbreviations: mM, millimolar; mL, milliliters; h, hour; TGP, total gas production; CO2, carbon dioxide; CH4, methane; H2, hydrogen; NC, nitro compounds; M. ruminantium M1, Methanobrevibacter ruminantium; M. stadtmanae, Methanosphaera stadtmanae; ENA, ethyl-nitroacetate; ENP, ethyl-2-nitropropionate; NO₃⁻, nitrate; SEM, standard error of the mean. Within each hour, means in the same row that do not share a common superscript letter are significantly different (*P* < 0.05). The absence of superscript letters within a row indicates no significant differences.

According to the results found in the combined experiment, doses of the NC significantly affected (*P* = 0.007) the log copy numbers of the total populations of methanogenic archaea (Table 4). Similarly, the combined experiment reflected significant changes (*P* < 0.001) in the log copy numbers of *M. stadtmanae* due to the effect of dose (Table 4), aligning with the observations obtained in the individual experiments. On a pairwise comparison, ENA significantly increased (*P* < 0.01) the log copy numbers of *M. stadtmanae* and decreased (*P* < 0.01) the log copy numbers of *M. ruminantium* compared with all other treatments at 12 and 24 h. In the combined experiment, the log copy numbers of *M. stadtmanae* increased by 27%, 6%, and 11% with 8 mM of ENA, 16 mM of ENP, and 12 mM of NO_3_^-^ at 24 h post-incubation, respectively, compared with the 0 mM dose. In the combined experiment, the log copy numbers of *M. ruminantium* M1 were overall lower in all nitro-treated cultures relative to the 0 mM dose (Table 4), consistent with the results observed in the individual experiments. At 24 h, the log copy numbers of *M. ruminantium* M1 decreased by 25% and 2% relative to the 0 mM dose at 8 mM of ENA and 16 mM of ENP, respectively. Finally, the log copy numbers of the methanogenic archaeon ISO4-H5 differed significantly for ENA (*P* < 0.001), ENP (*P* = 0.035), and NO_3_^-^ (*P* = 0.003) (Table 4). The log copies of this archaeon increased by 19, 5, and 8% relative to the 0 mM dose with 8 mM of ENA, 16 mM of ENP, and 12 mM of NO_3_^-^, respectively (Table 4), consistent with the results observed in the individual experiments.

Although the total VFA concentration did not differ in the individual experiments, these results differ from those obtained in the combined experiment, in which the total VFA concentrations in all the nitro-treated vials were significantly lower (*P* = 0.002) compared with the 0 mM dose at 24 h post-incubation (Table 5). In the combined experiment, ENA and ENP reduced acetate accumulations by 24% and 7%, respectively (Table 5), consistent with the results obtained in the individual experiments. Furthermore, ENA increased propionate concentrations by 10% but was 4 and 5% lower in ENP- and NO₃⁻-fermentation vials compared with the 0 mM dose (Table 5), consistent with the results obtained in the individual experiments. In this experiment, we also observed butyrate accumulations at 8 mM of ENA, which were 15% higher than the 0 mM dose at 24 h post-incubation (Table 5) and were similar to those of the individual experiments. The concentrations of isobutyrate in the vials treated with 8 mM of ENA and 16 mM of ENP were 16% and 10% lower at 24 h post-incubation relative to the 0 mM dose (Table 5), consistent with ENA and ENP’s adverse effects observed in the individual experiments.

**TABLE 5:** Antimethanogenic effects of different doses of NC on rumen fermentation parameters after 24 h of incubation^*a*^

VFA	Control (0 mM)	ENA (8 mM)	ENP (16 mM)	NO₃⁻ (12 mM)	SEM	*P*-value
Acetate (mM)	82.9^a^	62.8^b^	77.1^c^	80.4^d^	0.29	<0.001
Propionate (mM)	31.1^a^	34.1^b^	29.8^a^	29.6^a^	0.32	0.002
Butyrate (mM)	25.6^ab^	29.3^a^	25.8^ab^	22.4^b^	0.91	0.027
Valerate (mM)	3.05^a^	2.61^b^	3.01^a^	2.82^c^	0.02	<0.001
Isovalerate (mM)	3.49^ab^	3.58^a^	3.50^ab^	3.37^b^	0.02	0.010
Isobutyrate (mM)	1.69	1.43	1.52	1.46	0.21	0.817
Total VFA (mM)	148^a^	134^b^	141^c^	140^c^	0.91	0.002
Acetate (mol %)	56.0^ab^	46.9^c^	54.7^a^	57.4^b^	1.55	0.0001
Propionate (mol %)	21.0^a^	25.4^b^	21.1^a^	21.0^a^	0.72	0.0010
Butyrate (mol %)	17.3^a^	21.9^b^	18.3^a^	15.9^a^	0.85	0.0066
Valerate (mol %)	2.06^ab^	1.95^a^	2.13^b^	2.00^a^	0.03	0.0119
Isovalerate (mol %)	2.36^a^	2.67^b^	2.48^c^	2.39^ac^	0.05	0.0004
Isobutyrate (mol %)	1.14	1.07	1.08	1.04	0.05	0.9592

^*a*^ Data represent mean ± SEM from the comparison experiment. The selected doses represent optimal concentrations from initial screening: 8 mM (ENA), 16 mM (ENP), and 12 mM (NO₃⁻). VFA concentrations were measured by an HPLC system. Abbreviations: mM, millimolar; mol %, molar proportion; VFA, volatile fatty acid; NC, nitro-compounds; ENA, ethyl-nitroacetate; ENP, ethyl-2-nitropropionate; NO₃⁻, nitrate; SEM, standard error of the mean. Within a row, means without a common superscript letter are significantly different (*P* < 0.05). The absence of superscript letters within a row indicates no significant differences.

The molar proportions of individual VFAs also changed significantly across treatments. The acetate molar proportion decreased by 16% in ENA-treated vials and by 2% in ENP-treated vials but increased slightly (by 2%) in NO₃⁻-treated vials relative to the control (*P* = 0.0001). Conversely, ENA increased the molar proportion of propionate by 21% (*P* = 0.001), whereas ENP and NO₃⁻ showed no change relative to the control. Butyrate proportions increased by 27% in ENA-treated vials but decreased by 8% in NO₃⁻-treated vials (*P* = 0.007). Valerate and isovalerate proportions were also significantly affected (*P* < 0.05), with increases of 3%–13% in ENA- and ENP-treated vials compared with the control, whereas isobutyrate proportions remained unchanged (*P* > 0.05).

### Phase 2: Testing the NC on a pure culture of *M. stadtmanae*

#### Effect of the NC on the growth of *M. stadtmanae* and gaseous composition

The addition of NC to pure cultures of *M. stadtmanae* adversely affected its growth (Table 6). Cultures treated with ENA (*P* < 0.001) and ENP (*P* < 0.001) significantly suppressed OD_600_ at 24 and 48 h post-incubation compared with untreated cultures. Additionally, doses of NO₃⁻ above 0.75 mM had a similar suppressive effect (*P* < 0.001) as ENA and ENP (Table 6). TGP in ENA-, ENP-, and NO₃⁻-treated cultures was higher than in untreated cultures (Table 6). After performing TGP measurements at each sampling hour, the gaseous composition of all the cultures was analyzed. CO_2_ production in all ENA- and ENP-treated cultures was higher compared to untreated cultures. A similar effect was noted in NO₃^-^-treated cultures, although at doses above 0.75 mM (Table 6). Coupled to these results, H_2_ levels remained elevated in ENA, ENP, and in NO₃^-^ at doses above 0.75 mM, indicating low H_2_ consumption (Table 6). These findings point toward the heightened susceptibility of *M. stadtmanae* to ENA and ENP, even at low doses, and at doses above 0.75 mM of NO₃⁻. Furthermore, based on the gaseous composition analysis, a significant proportion of the gas present in the cultures’ headspace was not recovered. Gas recoveries ranged from 20% to 60% of the TGP of treated and untreated cultures, markedly lower than the corresponding TGP for each treatment.

**TABLE 6:** Growth inhibition and gas modulation of *M. stadtmanae* by different doses of NC at 24 and 48 h of incubation utilizing Hungate tube cultures: OD_600_, gas composition (mL), and proportions (%)^*a*^

		24 h							48 h							SEM	*P*-value		
Variable	Inhibitor	0 (mM)	0.125 (mM)	0.250 (mM)	0.50 (mM)	0.75 (mM)	1 (mM)	1.25 (mM)	0 (mM)	0.125 (mM)	0.250 (mM)	0.50 (mM)	0.75 (mM)	1 (mM)	1.25 (mM)		Dose	Hour	D x H
OD_600_	ENA	0.60^a^	0.12^b^	0.13^b^	0.13^b^	0.12^b^	0.13^b^	0.12^b^	1.22^a^	0.15^b^	0.20^b^	0.18^b^	0.20^b^	0.21^b^	0.18^b^	0.02	<0.0001	<0.0001	<0.0001
TGP (mL)		1.79	3.34	3.56	3.31	3.11	3.24	3.12	2.19	3.46	3.58	3.34	3.13	3.26	3.21	0.34	0.003	0.598	0.998
CO_2_ (mL)		0.09^a^	0.49^b^	0.49^b^	0.50^b^	0.49^b^	0.47^b^	0.49^b^	0.01^a^	0.47^b^	0.48^b^	0.48^b^	0.48^b^	0.44^b^	0.47^b^	0.02	<0.0001	0.004	0.617
CH_4_ (mL)		0.59^a^	0.00^b^	0.00^b^	0.00^b^	0.00^b^	0.00^b^	0.00^b^	1.06^a^	0.00^b^	0.00^b^	0.00^b^	0.00^b^	0.00^b^	0.00^b^	0.36	<0.0001	0.568	0.987
H_2_ (mL)		0.04	0.36	0.52	0.78	0.82	0.83	0.84	0.02	0.36	0.51	0.80	0.79	0.82	0.83	0.10	<0.0001	0.892	1.000
OD_600_	ENP	0.56^a^	0.14^b^	0.13^b^	0.16^b^	0.15^b^	0.14^b^	0.16^b^	1.10^a^	0.15^b^	0.15^b^	0.16^b^	0.16^b^	0.15^b^	0.17^b^	0.02	<0.0001	<0.0001	<0.0001
TGP (mL)		1.90	3.51	3.70	3.36	3.60	3.35	3.31	2.23	3.75	3.77	3.45	3.77	3.63	3.50	0.34	<0.0001	0.1907	0.9989
CO_2_ (mL)		0.09^a^	0.48^b^	0.47^b^	0.48^b^	0.49^b^	0.52^b^	0.52^b^	0.02^a^	0.46^b^	0.46^b^	0.48^b^	0.48^b^	0.51^b^	0.51^b^	0.02	<0.0001	0.0645	0.6364
CH_4_ (mL)		0.39^a^	0.00^ab^	0.00^ab^	0.00^ab^	0.00^b^	0.00^b^	0.00^b^	0.71^a^	0.00^ab^	0.00^b^	0.00^b^	0.00^b^	0.00^b^	0.00^b^	0.36	<0.0001	0.4622	0.2569
H_2_ (mL)		0.04	0.78	0.79	0.83	0.82	0.78	0.86	0.02	0.71	0.78	0.79	0.81	0.78	0.85	0.10	<0.0001	0.7145	1.000
OD_600_	NO3⁻	0.36^a^	0.24^ab^	0.21^ab^	0.12^b^	0.08^b^	0.14^b^	0.12^b^	0.91^a^	0.90^a^	0.89^a^	0.92^a^	0.11^b^	0.16^b^	0.16^b^	0.02	<0.0001	<0.0001	<0.0001
TGP (mL)		1.61	2.55	2.61	2.45	3.46	3.38	3.51	1.89	2.72	2.80	2.68	3.61	3.71	3.97	0.34	<0.0001	0.201	1.000
CO_2_ (mL)		0.06^a^	0.07^a^	0.08^a^	0.08^a^	0.39^b^	0.38^b^	0.38^b^	0.01^a^	0.01^a^	0.01^a^	0.01^a^	0.36^b^	0.35^b^	0.36^b^	0.02	<0.0001	<0.0001	0.413
CH_4_ (mL)		0.30^a^	0.29^a^	0.33^a^	0.28^a^	0.00^b^	0.00^b^	0.00^b^	0.50^a^	0.49^a^	0.45^a^	0.45^a^	0.00^b^	0.00^b^	0.00^b^	0.36	<0.0001	<0.0001	0.530
H_2_ (mL)		0.03^a^	0.03^a^	0.03^a^	0.03^a^	0.55^b^	0.58^b^	0.61^b^	0.02^a^	0.02^a^	0.02^a^	0.02^a^	0.53^b^	0.56^b^	0.57^b^	0.10	<0.0001	0.211	0.999

^*a*^ Data represent mean ± SEM from pure culture experiments. The selected doses represent optimal concentrations from the initial screening: 0.1 and 3 mM for ENA, ENP and NO₃⁻. Gas composition values were derived from Hungate tube cultures. . Abbreviations: mM, millimolar; mL, milliliters; h, hour; D, dose; H, hour; NC, nitro-compound; ENA, ethyl-nitroacetate; ENP, ethyl-2-nitropropionate, NO₃⁻, nitrate; OD600, optical density measured at a wavelength of 600 nanometers; TGP, total gas production; CO2, carbon dioxide; CH4, methane; H2, hydrogen; SEM, standard error of the mean. Within each hour, means in the same row that do not share a common superscript letter are significantly different (*P* < 0.05). The absence of superscript letters within a row indicates no significant differences.

## DISCUSSION

This study represents a novel effort to characterize the effects of ENA, ENP, and NO₃⁻ on gaseous composition, rumen fermentation, and bacterial-archaea populations *in vitro*, followed by validation using a model rumen methanogenic isolate. It also lays the foundational framework for understanding the mechanisms underlying their potent antimethanogenic activities in the rumen.

Several noteworthy findings emerged from this study. First, the use of the ANKOM RF Gas Production System to simulate ruminal conditions, combined with subsequent analyses of gaseous composition, microbial populations, and VFAs, demonstrated strong reproducibility. This consistency was observed even when rumen inoculum was collected from the same lactating Holstein cow at different stages of her lactation cycle. Notably, the proportions of individual gases at 24 h post-incubation were highly consistent across untreated controls from five independent *in vitro* experiments, averaging 53% CO₂, 26% CH₄, and <1% H₂. In a previous study, we similarly reported that individual VFA proportions remain relatively constant within individual cows (30), suggesting that the relative proportions of fermentation gases may also be host-specific, potentially shaped by microbial composition and host genetics.

In the present study, CH₄ accounted for 26% of TGP in untreated controls across all *in vitro* experiments, consistent with previous reports (31, 32). ENA, ENP, and NO₃⁻ markedly reduced CH₄ to 0.09%, 0.01%, and 12% of TGP, respectively, confirming their potent antimethanogenic effects. Despite strong CH₄ inhibition by all three inhibitors, TGP remained largely stable, except for a modest reduction with ENP. In ENA-treated vials, increased H₂ accumulation appeared to offset CH₄ loss, maintaining TGP. NO₃⁻ treatment yielded slightly higher TGP, likely due to the formation of alternative gases such as N₂ or NH₃. Although the fate of spared H₂ was not quantified in this study, these findings suggest that each compound redirects reducing equivalents differently through direct inhibition of methanogens, altered H₂ flux, or diversion of electrons to alternative sinks such as VFA or nitrate reduction. Although this study provides foundational data on microbial, VFA, and gas shifts associated with NCs, further research is warranted to elucidate the mechanistic basis of these interactions, particularly those governing microbial cross-talk, H₂ fluxes, and fermentation pathways.

The addition of ENA to *in vitro* rumen incubations significantly altered gaseous composition, bacterial–archaeal populations, and VFA profiles. Most notably, CH₄ production was completely inhibited, dropping from 26% in controls to nearly zero, whereas H₂ concentrations increased markedly to ~27%. This observation indicates direct inhibition of hydrogenotrophic methanogenesis, supported by a 2-fold reduction in *Methanobrevibacter ruminantium*, a dominant hydrogenotrophic archaeon in the rumen (33, 34). Partial inhibition of *M. stadtmanae* and other methylotrophic archaea suggests that ENA’s antimethanogenic effect may be species-specific. Mechanistically, ENA likely inhibits hydrogenase uptake activity, thereby restricting H₂ consumption by archaea for CH₄ synthesis (10, 35–37). In the rumen, H₂ is both produced and consumed by microbes, but methanogens specialize in the latter (37). Thus, inhibition of H₂ uptake by ENA explains the substantial H₂ accumulation observed in ENA-treated fermentation vials.

If ENA inhibited methanogens by 100%, it is plausible that H₂ uptake was completely suppressed, theoretically yielding up to four times the volume of CH₄ as H₂. However, in the current study, the measured H₂ volume was approximately equal to that of CH₄, indicating that nearly three-quarters of the expected H₂ remains unaccounted for. The resulting increase in dissolved H₂ may have elevated ruminal partial pressure, which could have been sensed by H₂-producing bacteria (38), triggering feedback regulation to reduce H₂ and acetate production. Greening et al. (37) also reported that *Ruminococcus albus* and related H₂-producing species respond to elevated *P*[H₂] by switching from acetate- and H₂-producing fermentation toward more reduced end products such as succinate or ethanol. Although relative bacterial abundances remained unchanged in the 16S rDNA analysis, it may be speculated that the spared H₂ increased p[H₂], reducing acetate and H₂ output while possibly increasing unmeasured metabolites such as succinate or ethanol. Pitta et al. (39) further indicated that butyrate production can rise under inhibited methanogenesis due to chain elongation involving ethanol and VFAs, which may similarly explain the elevated butyrate observed in ENA-treated cultures.

The accumulated H₂ may therefore have acted as both a direct result of inhibited methanogenesis and a regulatory signal driving shifts in bacterial metabolism. This interpretation is consistent with the observed ~10% decline in acetate concentrations and increased propionate and butyrate levels in ENA-treated cultures. Collectively, these findings support the hypothesis that ENA-induced inhibition of H₂ uptake increased p[H₂], prompting feedback adjustments in fermentative bacteria that decreased H₂ and acetate formation while enhancing propionate and butyrate synthesis. Although these changes appear favorable for redox balance, minor reductions in TGP and total VFA production suggest mild inhibition of overall fermentation activity, warranting further *in vivo* validation.

Although ENA’s effects appear primarily driven by hydrogen accumulation and feedback regulation of fermentative bacteria, ENP exhibited a contrasting pattern. Although ENP supplementation resulted in complete CH₄ inhibition, it was accompanied by a modest H₂ increase (~3%). The limited H₂ accumulation suggests that ENP exerts its antimethanogenic effects indirectly, with H₂ likely redirected into alternative pathways or utilized by other microbial populations. These findings align with previous reports showing partial inhibition of methanogenesis by structururally similar NCs without significant H₂ buildup, likely due to microbial adaptation or the presence of alternative H₂ sinks (40, 41). No significant shifts in methanogenic archaeal populations were detected, indicating that ENP may have a less direct effect than ENA on archaeal activity and H₂ metabolism. The observed 16% increase in CO₂ production likely reflects reduced carbon flow toward CH₄, consistent with other antimethanogenic studies (42). Fermentation profiles shifted in a dose-dependent manner: acetate decreased slightly (2%–3%), whereas propionate and butyrate increased modestly (2% and 1% at 8 and 16 mM, respectively), along with minor increases in valerate (0.1%) and isovalerate (0.5%). At lower doses, reduced TGP and minimal H₂ accumulation suggest enhanced H₂ uptake by propionate-producing bacteria, whereas at higher doses, stimulation of butyrate-producing taxa such as Pseudobutyrivibrio, Moryella, and members of the Lachnospiraceae family may occur (43–45).

Unlike ENA, whose effects can be mechanistically inferred through H₂ accumulation and feedback regulation, ENP’s mode of action remains less clear. Multi-omics approaches could help elucidate these dose-dependent shifts, including possible metabolic competition among microbial groups. Interestingly, total TGP and VFA concentrations declined slightly at 8 mM but increased at 16 mM, suggesting microbial adaptation or enhanced fermentation at higher doses. Overall, these results highlight ENP’s moderate antimethanogenic potential and its subtle influence on fermentation dynamics without significantly disrupting archaeal populations. Further *in vivo* studies are warranted to confirm these effects and evaluate their implications for rumen function and productivity.

In contrast to both ENA and ENP, the addition of NO₃⁻ led to moderate CH₄ inhibition, reducing CH₄ production by 50%, from 26% in controls to 12% at both low and high doses. Simultaneously, H₂ production declined from 0.34% to 0.2%, indicating that CH₄ suppression occurred through an indirect mechanism involving H₂ redirection rather than accumulation. Minimal changes in methanogenic archaeal populations further support this conclusion (46). These patterns likely reflect H₂ diversion toward NO₃⁻-reducing pathways that produce intermediates such as nitrite (NO₂⁻) and ammonia (NH₃), thereby limiting H₂ availability for methanogenesis (47). The dissimilatory reduction of NO₃⁻ to NH₃ is energetically more favorable (ΔG°′ = −600 kJ mol⁻¹) than the reduction of CO₂ to CH₄ (ΔG°′ = −136 kJ mol⁻¹), meaning that NO₃⁻ effectively competes with methanogens for electrons and redirects reducing power away from CH₄ synthesis toward NH₃ formation (48). With no notable disruptions in VFA profiles, the principal metabolic shift appears to be the redirection of H₂ utilization toward nitrate reduction (49). This mechanism differs fundamentally from ENA and ENP, as NO₃⁻ competes thermodynamically with CO₂ for reducing equivalents rather than altering microbial fermentation feedbacks. The absence of major fermentation impairment, combined with substantial CH₄ reduction, underscores NO₃⁻’s potential as a reliable antimethanogenic additive. Nevertheless, careful management is essential to prevent the accumulation of NO₂⁻ and NH₃ intermediates, which can pose toxicity risks to the host (50).

Using a pure culture model of *M. stadtmanae*, we further examined the NC-specific effects on methylotrophic methanogenesis. Similar to the *in vitro* rumen incubations, CH₄ inhibition was evident, with ENA causing marked H₂ accumulation (~27%) in both systems, confirming its direct inhibitory action. ENP induced lower H₂ accumulation in mixed cultures but reached comparable levels to ENA in pure culture, suggesting different interactions with microbial consortia. NO₃⁻ exhibited consistent behavior across systems, producing minimal H₂ accumulation and supporting its role in diverting H₂ toward nitrate reduction. All NCs strongly suppressed *M. stadtmanae* growth and CH₄ production, as reflected by low OD₆₀₀ values and negligible headspace CH₄. The growth and methanogenic activity of ENA- and ENP-treated cultures were severely impaired at all doses, whereas NO₃⁻ inhibition occurred primarily at concentrations above 0.75 mM. These results demonstrate the high sensitivity of *M. stadtmanae* to nitro compounds, consistent with *in vitro* observations, and emphasize the need to assess their effects on additional methanogenic species such as *M. ruminantium* M1 and the methanogenic archaeon ISO4-H5. Although 16S rDNA and RT-qPCR analysis of methanogenic archaea revealed no significant shifts, this may be due to the exclusive use of the solid fraction of *in vitro* samples, which may be more resilient to treatment.

Several limitations should be considered when interpreting these findings. First, *in vitro* batch culture systems cannot fully replicate the physiological complexity of the rumen, particularly regarding digesta flow, host-microbe interactions, and long-term microbial adaptation. Second, the use of rumen fluid from a single donor cow, although beneficial for experimental consistency, limits the generalizability of the results given known inter-animal variation in rumen microbiota and fermentation responses. Third, microbial analyses were performed only on the solid fraction and relied on taxonomic profiling, which does not capture functional gene expression or fully represent the liquid-phase microbial community. Additionally, CO₂ measurements obtained in this study likely do not reflect the true *in situ* concentrations, as bicarbonate buffering and gas-liquid exchange can influence CO₂ recovery. Nevertheless, CO₂ values were retained to provide context for understanding individual gas dynamics across treatments and guide future methodological refinements. As such, these data should be interpreted with caution, recognizing their relative rather than absolute accuracy. Future studies should include both solid and liquid fractions and integrate metagenomic or transcriptomic approaches, along with multi-donor inocula and *in vivo* validation, to better elucidate nitro-compound-mediated CH₄ mitigation and microbial adaptation. Despite these constraints, the high reproducibility across experiments supports the robustness of the findings and provides a solid foundation for further mechanistic and translational research.

### Conclusions

This study provides new insights into the distinct antimethanogenic mechanisms of ENA, ENP, and NO₃⁻ and establishes a foundation for their strategic use as targeted CH₄ mitigation agents. ENA emerged as the most potent inhibitor, directly suppressing hydrogenotrophic methanogenesis and triggering H₂ accumulation that redirected fermentation toward increased propionate and butyrate formation. ENP achieved complete CH₄ suppression through a subtler, dose-dependent modulation of fermentative pathways, whereas NO₃⁻ reduced CH₄ moderately by thermodynamically outcompeting methanogens for electrons without disrupting fermentation balance. Dose-response evaluations identified 8 mM ENA, 16 mM ENP, and 12 mM NO₃⁻ as optimal concentrations that effectively mitigated CH₄ while preserving rumen fermentation, providing physiologically relevant conditions for comparative assessment. Pure culture validation with *M. stadtmanae* confirmed compound-specific inhibition patterns, revealing differential sensitivities among methanogenic lineages. Collectively, these findings demonstrate the potential of nitro compounds as methane inhibitors and highlight the need for integrated multi-omics and *in vivo* validation to advance their application in sustainable ruminant production systems.

## Data Availability

The 16S rRNA raw sequence data have been submitted to the NCBI database under BioProject accession number PRJNA1262892.

## SUPPLEMENTAL MATERIAL

Material S1 aem.01033-25-s0001.docx Recipe to prepare media for *Methanosphaera stadtmanae*.

Table S1 aem.01033-25-s0002.docx Primers used in this study.

Table S2 aem.01033-25-s0003.xlsx Genus-level abundances.

Table S3 aem.01033-25-s0004.xlsx Spearman correlation.

Table S4 aem.01033-25-s0005.xlsx Spearman correlation p-values.

ASM does not own the copyrights to Supplemental Material that may be linked to, or accessed through, an article. The authors have granted ASM a non-exclusive, world-wide license to publish the Supplemental Material files. Please contact the corresponding author directly for reuse.
