# Supplementary material for: Investigating the antimethanogenic effects of selected nitro-compounds on methane production, rumen fermentation, and methanogenic archaea in vitro
Source: Appl Environ Microbiol. 2025 Nov 28;91(12):e01033-25. doi: 10.1128/aem.01033-25 (PMC12724261; doi:10.1128/aem.01033-25)
Supplement: Material S1 — Recipe to prepare media for Methanosphaera stadtmanae. [file aem.01033-25-s0001.docx]

**Supplementary Material S1**

**Recipe to prepare media for *Methanosphaera stadtmanae***

The *M. stadtmanae* DSM 3091 strain used was obtained from the Deutsche Sammlung für Mikroorganismen und Zellkulturen (Braunschweig, Germany). Based on its unique nutrient requirements and strict anaerobic nature, *M. stadtmanae* was cultured under specific media and incubating conditions to ensure its growth. The composition of the media for 1 L contained 200 mL of clarified rumen fluid, 2 g of trypticase peptone (BD-BBL), 2 g of yeast extract (BD-Difco), 0.5 g of sodium acetate, 0.5 g of sodium formate, 2 mL of a solution containing minerals and trace elements, 0.7 mL of a NiCl_2_ x 6 (H_2_O) solution at 0.1%, 3 mL of a FeSO_4_ x 7 (H_2_O) solution at 0.1%, 2.8 g of KH_2_PO_4_, 0.6 g of K_2_HPO_4_, 0.3 g of (NH_4_)2SO_4_, 1 g of NH_4_Cl, 0.6 g of NaCl, 0.15 g of MgSO_4_ x 7H_2_O, 0.08 g of CaCl_2_ x 2 (H_2_O), 0.5 mL of a sodium resazurin solution at 0.1% w/v, 4 g of NaHCO_3_, 2 mL of Wolin’s vitamin solution (10X), 0.5 g of L-Cysteine HCl x H_2_O, 20 mL of methanol at 50% v/v, 0.5 mL of a tungstate/selenite solution, and 773.3 mL of distilled water. It is worth mentioning that methanol was not added at this step but added to the Hungate tubes after autoclaving and during subculturing to protect its molecular structure. The media was mixed for 30 minutes to dissolve all the components completely. While mixing, the media was flushed with 100% CO_2_ gas for 30 min more, and while flushing, the pH was adjusted between 6.7 and 7.0.
