# Supplementary material for: Investigating the antimethanogenic effects of selected nitro-compounds on methane production, rumen fermentation, and methanogenic archaea in vitro
Source: Appl Environ Microbiol. 2025 Nov 28;91(12):e01033-25. doi: 10.1128/aem.01033-25 (PMC12724261; doi:10.1128/aem.01033-25)
Supplement: Table S1 — Primers used in this study. [file aem.01033-25-s0002.docx]

**Table S1.** Sequence-specific primers for methanogenic archaea.

| **Methanogenic archaea** | **Gene** | **Primer** | **Sequence (5’–3’)** | **Ta**  **(°C)** | **G+C^1^**  **(%)** | **Product**  **length (bps)** | **R^2^** |
| --- | --- | --- | --- | --- | --- | --- | --- |
| *Methanobrevibacter ruminantium* M1 | *mcrG* | Frd | CTGGAGCAAAGTACATGGAGTCTA | 56 | 55 | 130 | 0.9987 |
|  |  | Rev | CACCCACCTCTCGACGAACTC |  |  |  |  |
| *Methanosphaera stadtmanae* DSM3091 | *mtaB* | Frd | AAATACAGAGATCCTCAAGGTGTAA | 57 | 55 | 124 | 0.9897 |
|  |  | Rev | CTATTGCCTTAAGTGCTGCTG |  |  |  |  |
| *Methanogenic archaeon* ISO4-H5 | *mtbB* | Frd | CGTTGCAAAGAAGCTCGGAC | 55 | 60 | 89 | 0.9873 |
|  |  | Rev | GATACCAGTGCAGCTTCCGA |  |  |  |  |
| 16S rRNA | 16S rRNA | Frd | AATTGGAKTCAACGCCKGR | 56 | 61.1 | 148 | 0.9904 |
|  |  | Rev | TGTGTGCAAGGAGCAGGGAC |  |  |  |  |
